# Does Emotional Intelligence Buffer the Effects of Acute Stress? A Systematic Review

**DOI:** 10.3389/fpsyg.2019.00810

**Published:** 2019-04-17

**Authors:** Rosanna G. Lea, Sarah K. Davis, Bérénice Mahoney, Pamela Qualter

**Affiliations:** ^1^School of Psychology, College of Business, Psychology and Sport, University of Worcester, Worcester, United Kingdom; ^2^School of Environment, Education and Development, Institute of Education, University of Manchester, Manchester, United Kingdom

**Keywords:** emotional intelligence, stress, reactivity, recovery, affect, physiology, mood

## Abstract

People with higher levels of emotional intelligence (EI: adaptive emotional traits, skills, and abilities) typically achieve more positive life outcomes, such as psychological wellbeing, educational attainment, and job-related success. Although the underpinning mechanisms linking EI with those outcomes are largely unknown, it has been suggested that EI may work as a “stress buffer.” Theoretically, when faced with a stressful situation, emotionally intelligent individuals should show a more adaptive response than those with low EI, such as reduced reactivity (less mood deterioration, less physiological arousal), and faster recovery once the threat has passed. A growing number of studies have begun to investigate that hypothesis in respect to EI measured as both an ability (AEI) and trait (TEI), but results are unclear. To test the “stress-buffering” function of EI, we systematically reviewed experimental studies that explored the relationship between both types of EI and acute stress reactivity or recovery. By searching four databases, we identified 45 eligible studies. Results indicated that EI was only adaptive in certain contexts, and that findings differed according to stressor type, and how EI was measured. In terms of stress reactivity, TEI related to less mood deterioration during sports-based stressors (e.g., competitions), physical discomfort (e.g., dental procedure), and cognitive stressors (e.g., memory tasks), but did not appear as helpful in other contexts (e.g., public speaking). Furthermore, effects of TEI on physiological stress responses, such as heart rate, were inconsistent. Effects of AEI on subjective and objective stress reactivity were often non-significant, with high levels detrimental in some cases. However, data suggest that both higher AEI and TEI relate to faster recovery from acute stress. In conclusion, results provide mixed support for the stress-buffering effect of EI. Limitations and quality of studies are also discussed. Findings could have implications for EI training programmes.

## Introduction

The concept of emotional intelligence (EI) has generated a high level of public and scientific interest, and controversy, ever since its inception (Salovey and Mayer, 1990). EI is an umbrella term that captures how we perceive, regulate, use, and understand our own emotions and the emotions of others (Zeidner et al., 2009). Two competing conceptualisations of EI exist: trait EI (TEI) and ability EI (AEI). TEI refers to a collection of emotional perceptions and dispositions assessed through self-report questionnaires (Petrides et al., 2007). In contrast, AEI is concerned with emotion-related cognitive abilities, measured using maximum performance tests in a similar manner to IQ (Mayer et al., 2008). Because both TEI and AEI predict good health, successful relationships, educational attainment, and work-related success, among other positive life outcomes (Brackett et al., 2011; Petrides et al., 2016), higher levels are generally regarded as beneficial. However, key questions remain unanswered. We do not fully understand the mechanisms linking EI to those positive outcomes—*how* and *when* is EI useful? While cross-sectional studies are useful for indicating potential relationships between EI and outcomes, they do not explain how EI might help us handle everyday challenges. Furthermore, while the incremental validity of EI is promising in some cases (Andrei et al., 2016; Miao et al., 2018), there are concerns that EI may not predict other outcomes any better than related constructs, such as personality and cognitive ability (Schulte et al., 2004). Moreover, a growing literature also warns that EI may have an unhelpful “dark side” (Davis and Nichols, 2018). Given the substantial interest in training EI across the lifespan (e.g., Nelis et al., 2011; Ruiz-Aranda et al., 2012), it is imperative that we understand more about how EI works, and why it leads to its beneficial effects. To develop the “science” of EI, robust methodology is needed to assess how EI relates to automatic, unconscious emotional processing (Fiori, 2009).

### Significance of Acute Stress Reactivity and Recovery

One mechanism through which EI may lead to positive effects is by acting as a “stress buffer” (Mikolajczak et al., 2009). EI may minimize the (acute) stress experienced in demanding situations, or situations perceived as demanding. That hypothesis has been used to explain a wealth of adaptive findings across educational (e.g., transition to secondary school), clinical (e.g., suicidal behaviors), and occupational domains (e.g., burnout) (Day et al., 2005; Cha and Nock, 2009; Williams et al., 2009). When confronted with a stressor, individuals need to initiate a “fight or flight” response, and then shut off the response once the stressor ceases (McEwen, 2006). The *extent* to which an individual responds to the stressor—stress reactivity—is an important indicator of physiological and psychological functioning (Henze et al., 2017). However, stress researchers disagree on whether *hypo* (reduced) or *hyper* (elevated) reactivity is most adaptive in stressful situations (e.g., Phillips et al., 2013; Hu et al., 2016). Clearly, some reactivity (i.e., not entirely blunted) is necessary for survival. For non-clinical populations, however, hyperreactivity to acute stress is detrimental in most cases. In the short term, high levels of acute stress can impair clinical decision-making in health professionals (LeBlanc, 2009; Arora et al., 2010), and the performance of sports players (Van der Does et al., 2015; Rano et al., 2018). Hyperreactivity can also adversely impact memory task performance in controlled experimental settings (e.g., Kuhlmann et al., 2005; Tollenaar et al., 2009), though not always (Nater et al., 2006).

In the long term, dysregulated responses to everyday stressors can accumulate and cause “wear and tear” on the body (Chida and Hamer, 2008), which can sometimes manifest into psychosomatic pathology. For example, individuals can develop hypertension and atherosclerosis (Matthews et al., 2004; Heopniemi et al., 2007; Chida and Steptoe, 2010). How quickly people recover, or “bounce back,” from acute stress is another revealing aspect of the stress response (Linden et al., 1997). It is well-established that recovering faster from stressful experiences is more adaptive in most contexts (e.g., Burke et al., 2005; Geurts and Sonnentag, 2006), as this limits unnecessary exposure to the detrimental downstream effects of the “fight or flight” response (i.e., cortisol, cardiac activity, neural activation; McEwen, 2017). Taken together, evidence suggests that reduced reactivity, and faster recovery, can be thought of as the “adaptive” pattern of responding to an acutely stressful stimulus.

Because the stress pathway is complex, acute stress can be generated experimentally in many different ways. Common methods include the Velten technique (where participants read self-evaluative statements, such as “I'm discouraged and unhappy about myself”; Velten, 1968), or presenting participants with emotive video clips (e.g., Ramos et al., 2007). Other methods are more performance-based. Participants can be instructed to prepare and deliver an impromptu speech (e.g., the “gold standard” Trier Social Stress Test; TSST; Kirschbaum et al., 1993). While the above procedures typically take place in the laboratory, some experiments use naturalistic stressors, such as an examination, or a competition (e.g., Lane et al., 2009). The specific emotions and physiological outcomes that emerge in a challenging situation are highly idiosyncratic, and depend on many stressor characteristics (i.e., levels of social evaluative threat, cognitive effort required; Denson et al., 2009). This makes synthesizing findings from studies that have induced stress differently is challenging.

In addition to acute stress induction, researchers also disagree on how best to *measure* our responses to acute stressors. The full body response to stress involves both arousal of the autonomic nervous system (ANS), and the somewhat faster HPA axis, in addition to the subjective experience (e.g., Baumann and Turpin, 2010). Measurements can be broadly considered as either (1) “physiological”; endocrine (e.g., cortisol) and ANS activity (e.g., heart rate, electrodermal activity, EEG), or (2) “psychological”; individual's perceptions of their mental state, assessed via self-report questionnaire. While the former, objective measures are free from self-report biases, the latter, subjective measures are also needed for context. For example, an increase in heart rate can result from both negative (e.g., fear) and positive (e.g., excitement) mental states (Lane et al., 2009). Largely due to practicality, however, many studies focus only one aspect of the stress response (i.e., objective or subjective stress), and rarely consider more than one neuroendocrine system (i.e., ANS or HPA-axis reactivity) (Campbell and Ehlert, 2012).

### Acute Stress Responding: A Role for EI?

Researchers are increasingly turning to EI in the search for individual differences that influence stress responding (Matthews et al., 2017). If EI is adaptive in stressful situations, high EI scorers should resond more in line with the adaptive profile (reduced reactivity, faster recovery), compared to low EI scorers. Much research so far has been correlational and/or cross-sectional, often restricted to questionnaire-based studies that test for associations between EI and dispositional stress. In most instances, higher levels of EI, especially TEI, correspond with lower levels of perceived occupational or life stress (e.g., Mikolajczak et al., 2006; Extremera et al., 2007). However, to substantiate claims of EI as a stress buffer, the process needs to be demonstrated “in action,” using controlled, experimental stress paradigms. While responses to laboratory-induced stress are not of clinical importance on their own, they represent the way that individuals ordinarily respond to everyday challenges, which has implications for adaptation (Henze et al., 2017). Identifying the types of stressful situations in which EI relates to stress responding is the next step in helping us to understand how EI works.

TEI and AEI are conceptually distinct (Pérez et al., 2005), supported by the weak correlations between self-report questionnaires and objective testing for EI (e.g., Brackett et al., 2006; Brannick et al., 2009). Generally, TEI is more strongly linked to adaptive outcomes than AEI (Harms and Credé, 2010; Martins et al., 2010). However, one school of thought suggests that TEI and AEI may work *together* to achieve positive life outcomes (e.g., Davis and Humphrey, 2012). Emotional skills (AEI) may be insufficient on their own. Individuals must also feel confident in those skills (TEI) for them to translate into behavior (Keefer et al., 2018). TEI and AEI may therefore influence stress-related processes differently, or be useful in different contexts. We might expect TEI to be especially useful for buffering reactivity in cognitive or psychosocial stress tasks, based on findings from experimental stress studies concerning self-efficacy, self-esteem, and happiness- positive traits that TEI maps onto (e.g., O'Donnell et al., 2008; Panagi et al., 2018). Research on AEI and stressor-activated processes is comparably scarce. However, links between AEI and selection of adaptive coping strategies (Davis and Humphrey, 2012) could suggest a role for AEI in stress reactivity and recovery. Constructs allied to AEI, such as emotion regulation ability, have also been linked to more adaptive affective responses to acute stress (e.g., Krkovic et al., 2018), but the role of other AEI competencies, as measured according to the AEI model (e.g., emotion perception; emotion understanding), are relatively unexplored. Besides EI conceptualization, other methodological factors are important to consider when determining the role of EI in stress processes. It is necessary to consider how studies induce stress, and how they measure stress reactivity and recovery.

### The Current Review

To test the hypothesis that EI buffers the effects of acute stress, all relevant experimental studies need to be systematically sourced and evaluated. The primary aim of the present systematic review, is, therefore, to identify emerging patterns regarding EI and stress reactivity and recovery in experimental studies. In particular, we aim to highlight types of stressful situation in which EI might be especially pertinent. Second, the review aims to examine aspects of methodological variation upon which the relationship between EI and reactivity may depend: EI measurement (TEI; AEI), stress induction paradigms, and stress measurement. Study quality will also be assessed to identify any common methodological study limitations.

## Methods

### Search Strategy

This review followed the PRISMA protocol (Moher et al., 2009). PsycInfo, MEDLINE, CINAHL, Academic Search Complete were searched exhaustively for studies investigating EI and stress reactivity. The term *emotional intelligence* was used, combined with any of the following keywords: stress, mood, affect, emotion^*^state, emotion regulat^*^, coping, heart rate, heart rate variability, blood pressure, cortisol, skin conductance, electrodermal activity, EEG, reactivity, or *recovery*. Reference lists of full text articles were also manually searched. Searches focused on studies published between 1990 and the present day, to correspond with the advent of Salovey and Mayer's paper where the EI concept was first introduced into the scientific psychological literature (Salovey and Mayer, 1990). Database searching took place during July 2018.

### Eligibility Criteria

**Figure 1:**
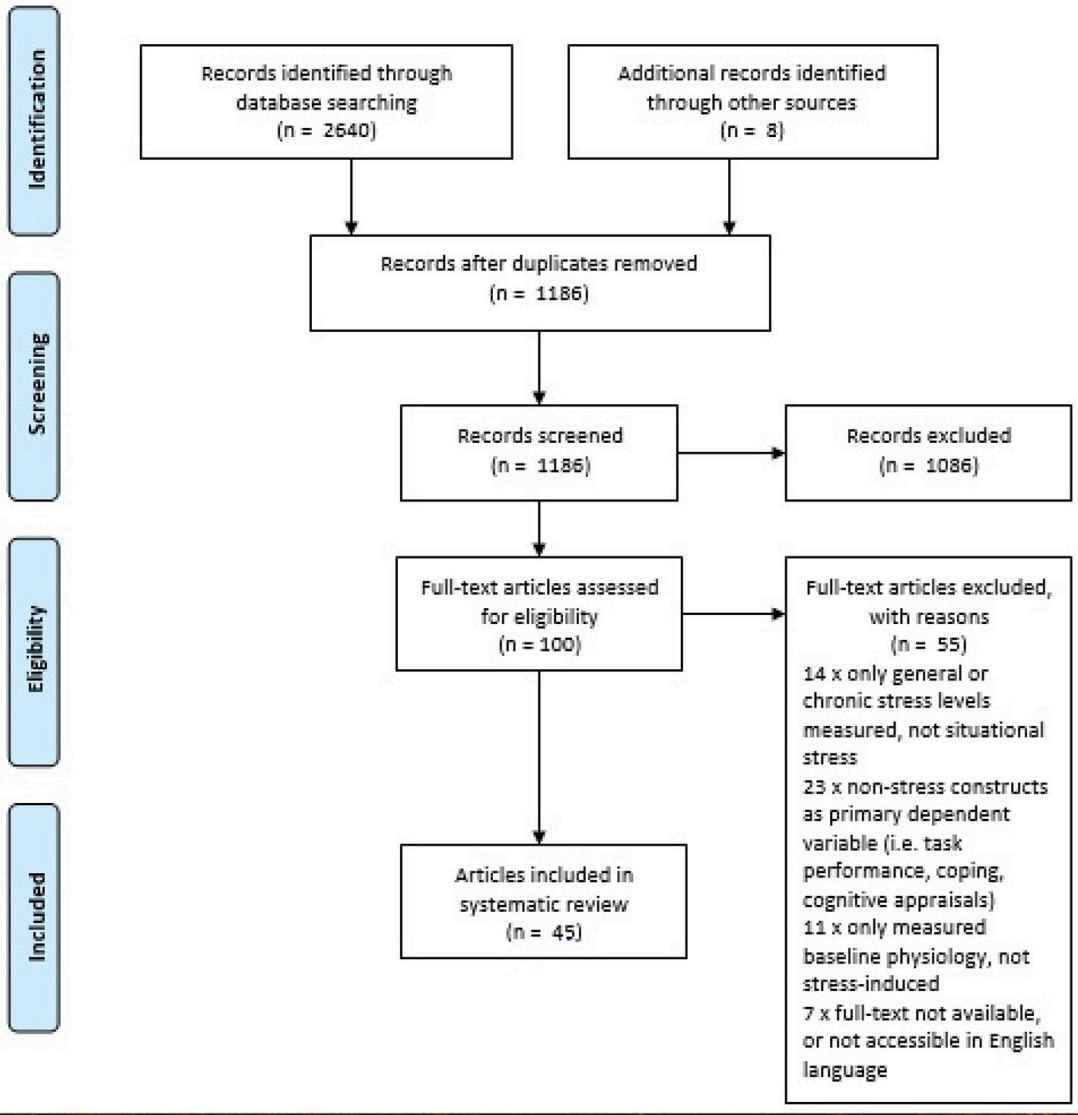
PRISMA 2009 flow diagram of search results (Moher et al., 2009).

Studies were included in the review if they met four inclusion criteria. First, only primary empirical quantitative research was included (i.e., not reviews, theoretical papers, or meta-analyses). Second, articles were required to define and measure EI explicitly using established models of EI, rather than just a single related facet (e.g., emotion regulation). We focused on overall EI to represent how EI is typically conceptualized with relation to life outcomes (Brackett et al., 2011), and within training programmes (e.g., Nelis et al., 2011). Examples of commonly used, acceptable TEI measures include the Trait Emotional Intelligence Questionnaire (TEIQue; Petrides, 2009), comprised of emotionality, sociability, self-control, and wellbeing subscales, and the Trait Meta Mood Scale (TMMS; Salovey et al., 1995), formed of clarity, repair, and attention subscales. Fewer AEI measures are available, the most popular tool being the Mayer-Salovey-Caruso Emotional Intelligence Test (MSCEIT; Mayer et al., 2002), which provides a four-branch assessment: perceiving emotions, using emotions to facilitate thought, understanding emotions, and managing emotions. Third, the outcome of interest was restricted to *acute* stress reactivity (i.e., a response to a situational stressor or mood induction). Outcomes could be either psychological (e.g., self-reported negative affect, or perceived stress), or physiological (e.g., cortisol, HR, EDA), or a combination. Fourth, participants were limited to non-clinical populations, to counteract the confounding influence that clinical symptomology can have on the stress response (Burke et al., 2005; De Rooij et al., 2010), but the participant sample could be of any age. Articles were also required to be available in full, and in the English language. If articles were unavailable, authors were contacted to request access. Studies were excluded if they did not meet all criteria. Many studies were excluded because they only included a measure of general perceived stress (e.g., work stress, life stress), rather than stress levels following a stress manipulation, or because they measured outcomes other than stress reactivity or recovery (e.g., task performance, coping). The first and second author independently screened the abstracts for suitability, and no inclusion discrepancies were identified. For details of the screening and selection process, see the PRISMA flow chart (Figure 1). Individual studies were appraised using an adapted version of the Effective Public Health Practice Project Quality Assessment Tool for Quantitative Studies (Table 1; Effective Public Health Practice Project, 1998), owing to its excellent psychometric properties (e.g., Armijo-Olivo et al., 2012).

**Table 1:** Adapted EPHPP tool for methodological quality of studies (Effective Public Health Practice Project, 1998).

**Component**	**Description**
Study design	Refers to whether studies comprised discrete control/experimental groups, and whether allocation to these was randomized
Confounders	Refers to whether authors controlled for confounding variables in the study design or analyses, and whether groups are balanced with respect to confounders
Data collection methods	Refers to whether measures used were reliable and valid
Analysis appropriate to question	Refers to whether statistical methods were appropriate for the study design and research question

*Other components of the EPHPP were removed as they were not applicable to the studies included in the review*.

## Results

### Study Characteristics

**Figure 2:**
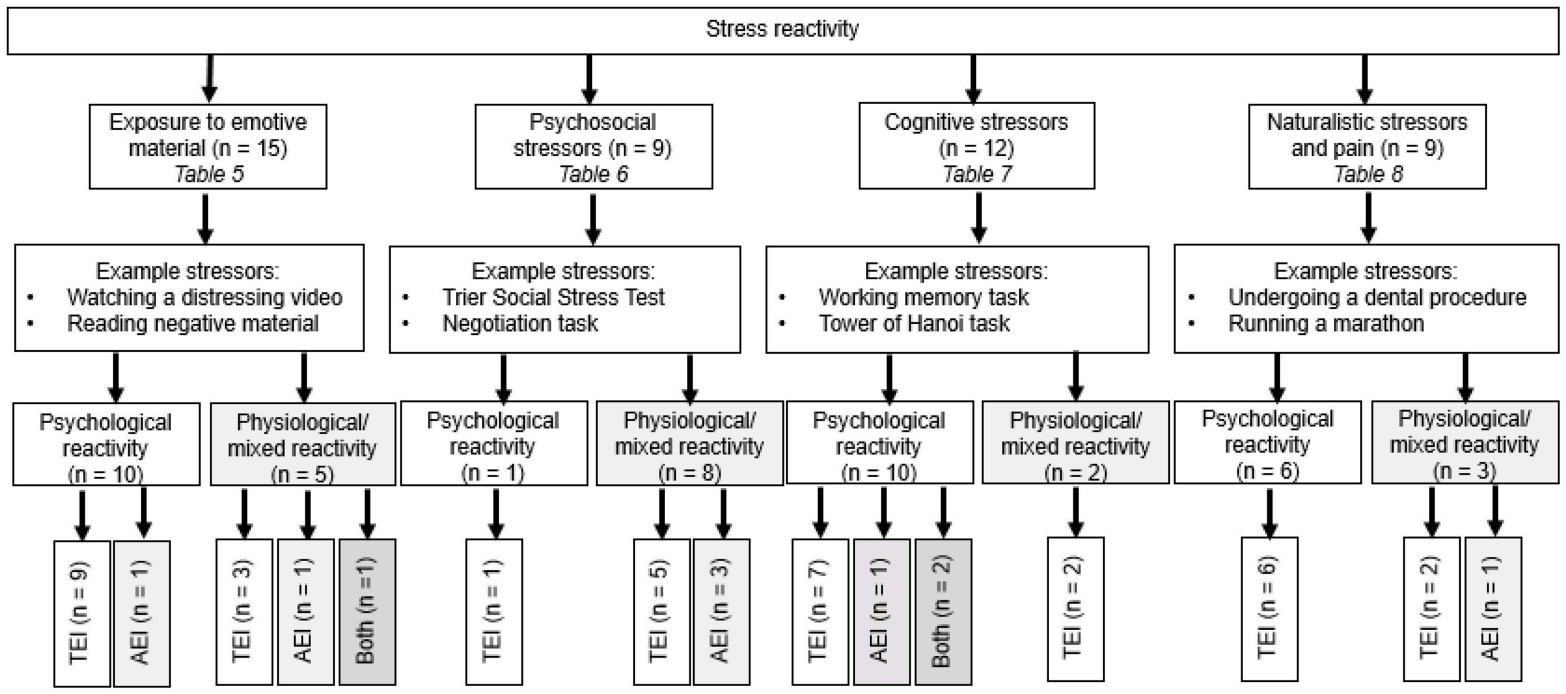
Overview of stress reactivity studies included in the review. AEI, ability emotional intelligence; TEI, trait emotional intelligence; both, measurement of both TEI and AEI; psychological stress reactivity, subjective measurements of reactivity (e.g., affect, mood, self-reported stress); physiological/mixed stress reactivity, objective measurements of stress reactivity (e.g., heart rate, cortisol, electrodermal activity), either alone or used alongside a psychological measure.

The search identified 40 papers (45 studies) for inclusion in the review. Publication location spanned 14 countries. Of the included studies, 42 used an adult sample, most of which consisted of university undergraduate students. Only three studies conducted research with younger populations: one with adolescents ages 13–15 years, and two with children ages 7–12 years. Studies varied in terms of EI instrumentation, stress induction procedure, and stress measurement. Figure 2 provides an overview of the stress reactivity studies identified.

#### EI Instruments

Thirty-four studies (78%) measured TEI, seven (16%) measured AEI, but only three (7%) measured both TEI and AEI. The TEIQue and MSCEIT were the most common tools for assessing TEI, and AEI, respectively. Half of the studies explored contributions from EI from a subscale level, in addition to the global score. Three studies by Papousek et al. (2008, samples 1 and 2; 2011) used only select subscales from a TEI measure (Self Report Emotional Ability Scale; SEAS: “perception of the emotions of others” and “regulation of one's emotions”). Table 2 details the breadth of tools utilized to measure EI across the review.

**Table 2:** EI measurement tools used in the review.

**EI type**	**Scale**	**Number of studies using**
Trait	Trait Emotional Intelligence Questionnaire Full or Short Form (TEIQue; Petrides, 2009)	16
	Schutte Emotional Intelligence Scale (SEIS)/Schutte Self-Report Emotional Intelligence Test (SSEIT; Schutte et al., 1998)	8
	Trait Meta Mood Scale (TMMS; Salovey et al., 1995)	6
	Self-Report Emotional Ability Scale (SEAS; Freudenthaler and Neubauer, 2005)	3
	Bespoke questionnaires using items from multiple TEI instruments	3
	Bar-On Emotional Quotient Inventory (Youth Version) (EQi-YV; Bar-On and Parker, 2000)	2
	Swinborne University Emotional Intelligence Test (SUEIT; Palmer et al., 2001)	1
Ability	Mayer-Salovey-Caruso Emotional Intelligence Test (MSCEIT; Mayer et al., 2002)	7
	Audio-Visual Test of Emotional Intelligence (AVEI; Zysberg et al., 2010)	1
	Situational Judgement Test of Emotional Abilities (Roberts et al., 2013)	1
	Situational Test of Emotion Management (STEM; MacCann and Roberts, 2008)	1
	Situational Test of Emotion Understanding (STEU; MacCann and Roberts, 2008)	1

*Some studies measured EI using more than one instrument*.

#### Types of Stressors Used

As expected, methods of stress induction varied between studies (see Figure 2 for examples of stressors used). Fifteen (33%) of the 45 studies in the review used passive methods of mood induction, in which participants viewed, read, or listened to, emotive material, but were not required to actively perform a task. The remaining 30 (67%) studies used either cognitive tasks (12 studies), psychosocial stress (9 studies), or more naturalistic stressors, such as a sporting task (6 studies) or physical discomfort (3 studies).

#### Stress Measurement

Twenty-nine studies (64%) examined subjective (self-reported) reactivity, eleven examined objective (physiological) reactivity (24%), and six examined both types of reactivity within the same experiment (12%). Generally, participants' acute psychological stress was conceptualized as the change in negative affect (NA) from baseline, for which the most popular mood assessment tool was the Positive and Negative Affect Schedule (PANAS; Watson et al., 1988), selected by 11 studies. Physiological stress was measured in a number of ways, including: cardiac measures (10 studies), cortisol secretion (6 studies), electro-dermal activity (EDA) (4 studies), or EEG (1 study). Depending on whether EI was conceptualized as a categorical or continuous variable, the principal measure was either the difference in mean reactivity/recovery between high-EI and low-EI individuals, or the relationship between EI and reactivity/recovery.

### Synthesized Findings

Studies in the review were principally classified according to the stressor context. Studies were further evaluated according to the type of EI model employed, and the type of stress reactivity assessment. Thus, the results section consists of: (1) Exposure to emotive material, (2) Psychosocial stress, (3) Cognitive tasks, and (4) Naturalistic stress and pain. Study findings relating to recovery from acute stress are considered separately (5). Because some studies explored multiple stress contexts, studies may appear under more than one heading. Where sufficient studies allowed, sections were further divided into subheadings: psychological reactivity, and physiological or mixed reactivity. Here, “mixed” refers to studies that included assessment of both psychological and physiological reactivity.

#### 1. Exposure to Emotive Material (Table 3)

Neither TEI nor AEI robustly predicted reactivity when exposed to visual, mentally recalled, or written emotive material. Some TEI studies indicated that TEI increased reactivity, but others found a negative relationship. AEI did not significantly predict reactivity in either direction.

**Table 3:** Studies that measured EI and reactivity during exposure to emotive material.

**Study**	**Sample**	**EI tool**	**Stressor**	**Outcome variable(s)**	**Quality**	**Relevant findings**
Ciarrochi et al., 2001	131 adolescents (58 females). *M* = 13.8, SD = 0.74	SEIS	Watching emotional video clips	Mood ratings	Weak	TEI was not related to mood following the stressor.
Fernández-Berrocal and Extremera, 2006	155 university students (123 females). *M* = 22, SD = 2.66	TMMS	Watching emotional video clips	PANAS	Strong	Clarity subscale was positively correlated with reactivity to anger mood induction, but also with recovery from sad mood induction. Repair was associated with greater affective balance, and faster recovery in all mood conditions.
Gohm, 2003, Study 1	250 university students (123 females). Mdn = 18.36	TMMS; MAS; AIM; EIS (scales from all indexed to form four “clusters” of participants	Recall of emotional event	Life event inventory reactions form	Moderate	“Hot” individuals (scoring high on Attention, Intensity, Clarity) were more reactive, and showed a more delayed recovery, than the other three types of individuals (“Overwhelmed,” “Cool,” “Cerebral”), in all mood conditions.
Study 3	236 university students (113 females). Mdn = 18	TMMS; MAS; AIM; EIS scales from all indexed to form four “clusters” of participants	Recall of emotional event	Life event inventory reactions form	Moderate	“Hot” individuals (scoring high on Attention, Intensity, Clarity) were more reactive than the other three types of individuals (“Overwhelmed,” “Cool,” “Cerebral”), in all mood conditions.
Laborde et al., 2011	30 handball players (all male). *M* = 22.5, SD = 1.7	TEIQue	Listening to sport competition-like stressor (e.g., crowd hissing, negative imagery)	HR variability	Moderate	TEI was negatively correlated with HR variability.
Limonero et al., 2015	64 undergraduate students (50 females). *M* = 22.32, SD = 4.3	MSCEIT	Viewing images from IAPS	STAI-S; POMS	Moderate	Emotion Facilitation and Emotion Understanding positively correlated with mood recovery, but were unrelated to reactivity. Emotion Perception and Management branches had no effect on reactivity or recovery.
Papousek et al., 2008, Sample 1	67 students (all female)	SEAS	Viewing emotional video clips	Affective rating scales for cheerfulness and happiness	Weak	Three-way interaction between Perception, Regulation and condition on affect. Together, low Perception and high Regulation were associated with reduced reactivity to the sad film, whereas high Perception and low Regulation were associated with increased reactivity to the cheerful film.
Papousek et al., 2008, Sample 2	56 students (all female). *M* = 23.9, SD = 4.4	SEAS	Viewing emotional video clips	HR; HRV	Weak	Three-way interaction between Perception, Regulation and condition on HR. Together, low Perception and high Regulation were associated with weak physiological responses to the sad film, whereas high Perception and low Regulation were associated with strong physiological responses.
Papousek et al., 2011	86 adults (42 females). *M* = 23.5, SD = 4.8	SEAS (Perception and Regulation only)	Listening to emotional sound clips	EEG (changes in dorsolateral asymmetry in the PFC used as an indicator of emotional arousal)	Weak	Three-way interaction between Perception, Regulation, and mood condition, on changes in PFC asymmetry. After watching the anxiety-inducing clip, only individuals with high Perception and high Regulation showed the expected pattern (a shift of PFC asymmetry to the right, followed by recovery to baseline). Individuals low on both Perception and Regulation showed most pronounced atypical responses (a shift to the left).
Petrides and Furnham, 2003	Study 2: 30 psychology undergraduates (22 females). *M* = 20.69, SD = 2.95	TEIQue	Viewing emotional video clips	POMS	Moderate	TEI correlated positively with reactivity to the sad mood induction.
Ramos et al., 2007	144 students (all females). *M* = 19.5, SD = 2.8	TMMS	Watching a video depicting sexual assault on two consecutive days	POMS- short form	Weak	Clarity correlated negatively with reactivity, and higher Repair was related to adaptation to the stressor.
Rash and Prkachin, 2013	56 university students (28 females). *M* = 26.38, SD = 9.36	MSCEIT	Personal recall paradigm	HR; RSA	Weak	The emotion perception scale was positively associated with cardiac reactivity when re-experiencing sadness, and promoted recovery.
Schutte et al., 2002, Study 3	47 students (35 females). *M* = 37.44, SD = 14.01	SEIS	Velten mood induction (reading statements about mood and self-worth)	PANAS	Moderate	TEI was associated with reduced PA deterioration after reading the negative statements.
Sevdalis et al., 2007, Study 1	60 students (43 females). *M* = 25.24, SD = 9.69	TEIQue-SF	Recall of negative life decision	PANAS	Weak	TEI negatively correlated with PA and positively with NA following the stressor.
Zysberg, 2012	84 university students (66 females). *M* = 23.69, SD = 1.79	SEIS; AVEI	Viewing emotional images	Subjective emotional responses; EDA	Weak	AEI predicted reduced EDA responses to both positive and negative stimuli. TEI did not predict EDA responses, but was negatively correlated with subjective emotional responses NB. Subjective responses were negatively correlated with EDA responses.

*SEIS, Schutte Emotional Intelligence Scale; TMMS, Trait Meta Mood Scale; DSSQ, Dundee Stress State Questionnaire; PANAS, Positive Negative Affect Schedule; PA, positive affect; NA, negative affect; MAS, Mood Awareness Scale; AIM, Affect Intensity Measure; EIS, Emotional Intensity Scale; BRUMS, Brunel Mood Scale; TEIQue-SF, Trait Emotional Intelligence Questionnaire—Short Form; WLEIS, Wong and Law Emotional Intelligence Scale; TAS-20, Twenty-Item Toronto Alexithymia Scale; MAS, Mood Awareness Scale; PANAS-X, Positive Affect Negative Affect Schedule—Expanded Form; POMS, Profile of Mood States*.

##### Psychological reactivity

The relationship between TEI and psychological stress was assessed in many studies. TEI increased reactivity when watching a holocaust documentary (Petrides and Furnham, 2003), and an apartheid clip (Fernández-Berrocal and Extremera, 2006). However, only the clarity subscale of the TMMS (which represents a perceived ability to discriminate clearly between emotions) was significant. Similarly, when participants were asked to recall a regrettable life decision, high TEI individuals presented a stronger emotional reaction (Sevdalis et al., 2007, Study 1). TEI also decreased reactivity in some cases, however. Ramos et al. (2007), Zysberg (2012), and Schutte et al. (2002, study 3) showed that high TEI scorers were less reactive to emotive video, images, and negative written statements, respectively. The only study to use an adolescent sample in the review (Ciarrochi et al., 2001) found no relationship between TEI and mood changes while watching a negative film.

Findings were more complicated when studies considered TEI “profiles”- differing levels of multiple subscales, rather than global TEI or single subscales. Papousek et al. (2008, sample 1) found that individuals scoring low on emotion perception, but high on emotion regulation, showed reduced psychological reactivity after viewing a sad emotional video clip. The reverse pattern was found for high perception but low on regulation. In essence, individuals who could perceive their emotions accurately, but not regulate them, were affected by the sad film to a greater extent. Gohm (2003, study 1) took a different approach, combining items from several TEI scales to form four “clusters” of participants (“hot,” “overwhelmed,” “cool,” “cerebral”). Of those clusters, “Hot” individuals (scoring high on attention, intensity, and clarity) were more reactive than the three other types when recalling an emotional event. That finding was replicated in a subsequent study (Gohm, 2003, study 3).

Two studies examined links between AEI and psychological reactivity to emotional images. In both cases, AEI had no effect on responses (Zysberg, 2012; Limonero et al., 2015).

##### Physiological or mixed reactivity

As before, findings were complex when TEI profiles were considered. When viewing sadness-inducing video clips, individuals scoring high on emotion perception, but low on emotion regulation subscales, showed increased cardiac reactivity (Papousek et al., 2008, sample 2). In contrast, low perception and high regulation scorers showed reduced reactivity. The same research group (Papousek et al., 2011) also found a relationship between subscales of the TEI and EEG outputs. After watching an anxiety-inducing clip, only those with both high emotion perception and high emotion regulation showed the expected EEG pattern (a shift of PFC asymmetry to the right). Individuals with low scores on these branches showed the most pronounced atypical response (a shift to the left), suggesting greater emotional arousal and poorer emotional regulation. Rash and Prkachin (2013) instead focused on AEI and physiological reactivity to recalling a sad memory. During the recall, individuals scoring highly on the perceiving emotion branch of AEI showed more extreme increases in HR than their low scoring counterparts.

Only one study (Zysberg, 2012) examined the role of both TEI and AEI in the context of both psychological and physiological reactivity. Findings identified different roles for TEI and AEI. When viewing negatively-valenced images, AEI (but not TEI) buffered EDA reactivity, whereas TEI (but not AEI) buffered emotional responses.

#### 2. Psychosocial Stress (Table 4)

Studies in this section induced stress through social evaluation. Most stressors were based on the highly standardized TSST protocol, where participants perform public speaking and mental arithmetic tasks in front of an audience (Kirschbaum et al., 1993). No clear pattern emerged concerning TEI and reactivity to psychosocial stressors. Though studies were limited in number, physiological reactivity appeared to *increase* as a function of overall AEI.

**Table 4:** Studies that measured EI and reactivity to psychosocial stress.

**Study**	**Sample**	**EI tool**	**Stressor**	**Outcome variable(s)**	**Quality**	**Relevant findings**
Bechtoldt and Schneider, 2016	157 university students (all male). *M* = 21.20, SD = 3.2	MSCEIT	TSST	Cortisol secretion; basal testosterone	Moderate	AEI was positively correlated with cortisol reactivity, but this effect was moderated by basal testosterone levels.
Laborde et al., 2014	28 near-expert tennis players (13 females). *M* = 23.88, range = 16–36	TEIQue	Tennis serving task, then arithmetic task from TSST	Cortisol secretion	Moderate	TEI was negatively correlated with overall cortisol secretion during the task.
Ling et al., 2018	156 adults (all male). *M* = 35.72, SD = 8.61	MSCEIT	Preparing and delivering a speech	SCL; HR	Moderate	Skin conductance level was positively associated with overall AEI.
Mikolajczak et al., 2009 Study 3	56 students (all male). *M* = 20.18, SD = 2.02	TEIQue	TSST	PANAS; cortisol secretion	Strong	TEI was associated with less self-reported mood deterioration and reduced cortisol secretion.
Salovey et al., 2002, Study 2	60 psychology students (all female)	TMMS	TSST	Cortisol; POMS	Moderate	Clarity was associated with reduced mood reactivity, but reduced cortisol secretion. Attention was positively associated with habituation to stressors.
Salovey et al., 2002, Study 3	48 psychology students (27 females). Age range = 17–23	TMMS	Achievement condition (arithmetic test and recitation of difficult passage) or interpersonal condition (social rejection paradigm)	Cortisol; BP	Moderate	Attention was positively associated with attenuated cortisol and systolic BP reactivity.
Schneider et al., 2013	126 psychology students (76 females). *M* = 20, SD = 4.6	MSCEIT	Arithmetic and speech tasks	PANAS; cardiac output; total peripheral resistance (indicator of BP)	Moderate	Emotion understanding was associated with higher PA and lower NA following the task, but in males only. In females, Emotion Management was associated with greater physiological reactivity.
Sevdalis et al., 2007, Study 2	24 adults (14 females). *M* = 22.21, SD = 2.81	TEIQue-SF	Failed negotiation task	Post-negotiation regret and disappointment	Weak	TEI was not related to immediate affect following the failed negotiation, but was negatively associated with regret and disappointment experienced 5 days later.
Thomas et al., 2018	110 males. *M* = 46.9, SD = 10.4	TEIQue	TSST-Group version	Cortisol; HRV	Weak	High TEI individuals showed greater cortisol (but not HRV) reactivity than low TEI individuals. TEI did not influence physiological recovery.

*Mikolajczak et al. (2007) was not included, as that study's data is reported in Mikolajczak et al. (2009) Study 3. TEIQue, Trait Emotional Intelligence Questionnaire; HR, heart rate; TSST, Trier Social Stress Test; SEAS, Self-Report Emotional Ability Scale; EEG, electroencephalogram; PFC, prefrontal cortex; SCL, skin conductance level; TMMS, Trait Meta Mood Scale*.

##### Psychological reactivity

In a small-sample study by Sevdalis et al. (2007, study 2, *n* = 24), participants took part in a negotiation task, where all failed by default. TEI failed to predict feelings of regret and disappointment, as assessed via two 5-point rating scales.

##### Physiological or mixed reactivity

Findings were inconsistent with regard to TEI and physiological reactivity. Mikolajczak et al. (2009, study 3)^1^ showed that TEI attenuated both cortisol reactivity and mood reactivity to the TSST. However, in a group version of the same task, Thomas et al. (2018) found the opposite: TEI predicted increased cortisol reactivity, but had no impact on HR. Another study showed that the TEI attention to emotion subscale (with items including, “I pay a lot of attention to how I feel”) exacerbated both cortisol and HR reactivity (Salovey et al., 2002, Study 3). With regards to AEI, higher levels represented greater cortisol secretion (Bechtoldt and Schneider, 2016) and EDA reactivity (Ling et al., 2018) to speech performances. Schneider et al. (2013) also focused on AEI, but with a particular emphasis on sex differences. Emotional understanding was associated with less mood deterioration in males, whereas emotion management was associated with greater cardiac reactivity in females.

#### 3. Cognitive Tasks (Table 5)

Stressors were classified as cognitive if they primarily assessed a mental process (e.g., attention, memory). Stress was typically induced from the difficulty of the task, and in some cases, it was impossible for the participant to perform well due to unrealistic time restraints, for example. The vast majority of these studies assessed the role of TEI, with most of those limited to psychological reactivity. While TEI buffered psychological reactivity in some computerized tasks, AEI was unrelated to reactivity.

**Table 5:** Studies that measured EI and reactivity to cognitive tasks.

**Study**	**Sample**	**EI tool**	**Stressor**	**Outcome variable(s)**	**Quality**	**Relevant findings**
Agnoli et al., 2015	66 adults (35 females). *M* = 24.1, SD = 2.2	TEIQue-SF	Bogus negative feedback in a timed helping behavior paradigm	PANAS	Moderate	Intensity of affective reaction to negative feedback did not differ by TEI.
Davis, 2018	161 adults (121 females). *M* = 25.24, SD = 8.81	TEIQue-SF STEM STEU	Failure task	PANAS	Strong	Experience of NA across the task did not vary as a function of either TEI or AEI.
Fallon et al., 2014	167 adults (110 female). *M* = 19.9, R = 18–33	TMMS; SJTEA	Decision-making task	DSSQ	Moderate	SJTEA (AEI) did not predict task distress, worry, or task engagement. However, task distress was significantly negatively correlated with clarity and repair subcscales. Task worry was negative correlated with the Clarity subscale.
Fellner et al., 2012	180 university students (111 females). *M* = 19.4, SD = 2.1	TMMS	Task discriminating terrorists from non-terrorists	DSSQ	Moderate	TEI was positively correlated with post-task distress, but not post-task worry.
Laborde et al., 2010	219 undergraduates (51 females). M(males) = 19.7 years, range = 18–25). M(females) = 19.5, range = 18–25)	TEIQue	A lecture, followed by a written examination	PANAS	Weak	TEI was positively correlated with PA and negatively with NA following the stressor.
Matthews et al., 2006	200 psychology students (132 females). *M* = 19.7, SD = 3.1	MSCEIT	High workload vigilance task; working memory task; impossible anagrams task	DSSQ	Strong	AEI was not related to reactivity in any of the tasks.
Matthews et al., 2015	129 psychology students (79 females). *M* = 20.8, SD = 3.8	TEIQue; WLEIS; TAS-20; MAS; TMMS (scales from all indexed to form 2 factors: clarity and attention)	Facial emotion perception tasks	DSSQ	Moderate	Clarity negatively correlated with post-task distress and worry. Attention was not related to reactivity.
Mikolajczak et al., 2009, Study 1	Study 1: 67 students (26 females). *M* = 21.23, SD = 2.01	TEIQue	Failure task (taken from Raven progressive matrices)	PANAS	Moderate	TEI negatively correlated with stress reactivity.
Study 2	62 students (47 females). *M* = 18.69, SD = 1.05	TEIQue	Failure task (taken from Raven progressive matrices)	PANAS; STAI		TEI marginally negatively correlated with stress reactivity.
O'Connor et al., 2016	225 adults (136 females). *M* = 23.54, range = 18–50	TEIQue-SF	Timed Tower of Hanoi	PANAS-X	Moderate	TEI indirectly predicted lower post-task NA via emotion-focused coping, but directly predicted greater post-task PA.
Pittarello et al., 2018	67 university students (53 female). *M* = 22.37, SD = 4.98	TEIQue-SF	Playing a computer game with an ethical dilemma component	HR; SCL	Weak	TEI was not related to HR or SCL reactivity.
Singh and Sharma, 2012	34 participants (all male). *M* = 24.4, SD = 3.2	SEIS	Playing a computer game with significant repeated defeats/constraints	SASRQ; HR; GSR; cortisol	Weak	TEI was not associated with HR or GSR responses, but was positively correlated with perceived stress during the task. Individuals with higher EI and low IQ had significantly higher post-stressor cortisol levels than other combinations of EI/IQ.

*EQ-I YV, Bar-On Emotional Quotient Inventory—Youth Version; TEIQue-SF, Trait Emotional Intelligence Questionnaire—Short Form; STAI, State Trait Anxiety Inventory; HR, heart rate; TSST, Trier Social Stress Test; PANAS, Positive Negative Affect Schedule; SEAS, Self-Report Emotional Ability Scale; HRV, heart rate variability; TMMS, Trait Meta Mood Scale; POMS, Profile of Mood States; BP, blood pressure; SEIS, Schutte Emotional Intelligence Scale; SASRQ, Stanford Acute Stress Reaction Questionnaire; GSR, galvanic skin response; SUEIT, Swinburne University Emotional Intelligence Test*.

##### Psychological reactivity

TEI buffered the effects of psychological stress in some cases. For example, global TEI score dampened the psychological stress induced by written examinations (Laborde et al., 2010). A similar pattern of findings also applied to multiple laboratory tasks. TEI predicted less mood deterioration following a facial perception task (Matthews et al., 2015), a mathematical puzzle (O'Connor et al., 2016), and a difficult IQ test (Mikolajczak et al., 2009, studies 1 and 2). In contrast, TEI was associated with increased distress during a terrorism-themed discrimination task (Fellner et al., 2012). In a computer game where participants received bogus negative feedback on a computerized task, TEI was unrelated to self-reported stress (Agnoli et al., 2015).

AEI was not significantly associated with psychological reactivity to a range of cognitive stressors, including tasks of working memory, vigilance, and impossible anagrams (Matthews et al., 2006). Two studies explored the role of both TEI and AEI in responding to cognitive stressors. The failure task paradigm employed by Davis (2018) indicated non-significant effects for both TEI and AEI on mood changes. However, Fallon et al. (2014) identified that effects of EI on reactivity to a decision-making task were dependent on EI type. While the clarity (e.g., “I am rarely confused about how I feel”) and repair (e.g., “I try to think good thoughts no matter how badly I feel”) subscales of the TMMS TEI measure predicted less psychological stress, AEI was unrelated to reactivity.

##### Physiological or mixed reactivity

TEI did not predict physiological reactivity in two studies that used a computer game to induce stress. On both occasions, EDA and HR reactivity was unrelated to TEI (Singh and Sharma, 2012; Pittarello et al., 2018). However, the latter study also considered TEI/IQ combinations, and found that a high TEI/low IQ combination was the most detrimental to cortisol reactivity. In that same study, TEI was associated with lower levels of perceived stress. Thus, while high TEI levels were protective on their own, they became harmful when paired with low IQ.

#### 4. Naturalistic Stress and Pain (Table 6)

Naturalistic stressors were defined as challenges that occurred naturally in the participants' everyday life (e.g., a sporting competition), or challenges that were generated to closely resemble such as situation. Evidence supported a protective role for TEI and AEI in stressful sports and pain-related contexts.

**Table 6:** Studies that measured EI and reactivity to naturalistic stressors.

**Study**	**Sample**	**EI tool**	**Stressor**	**Outcome variable(s)**	**Quality**	**Relevant findings**
Aminabadi et al., 2011	117 children (53 females). Age range = 7–12 years	EQ-I YV	Undergoing a dental procedure	Sound eye motor scale; modified dental anxiety scale	Moderate	During the dental procedure, TEI was related to less negative emotional behavioral responses, but not to self-reported anxiety.
Aminabadi et al., 2013	100 children (53 females). *M* = 8.48, SD = 1.41	EQ-I YV	Undergoing a dental procedure	Frankl behavioral rating scale; clinical anxiety rating scale	Moderate	Children with higher TEI were less anxious, and more cooperative, during the dental procedure.
Arora et al., 2011	16 medical students (6 females). *M* = 21.33, SD = 1.14	TEIQue-SF	Performing an unfamiliar surgical task (laparoscopy) in a virtual reality simulator	STAI; HR	Weak	TEI was positively correlated with self-reported stress, but also with faster recovery. TEI was not associated with HR during the task.
Lane et al., 2009	436 student athletes (223 females). *M* = 20.94, SD = 2.58	SEIS	Sporting competition; academic examination	BRUMS	Weak	TEI was associated with optimal mood states (i.e., vigor, low anger, low tension), but only for Appraisal of own Emotions, Optimism, and Utilization of Emotions subscales.
Lane et al., 2010	98 runners (23 females). *M* = 25.02, SD = 2.46	SEIS	10 mile running race	BRUMS	Weak	TEI predicted higher levels of pleasant post-race emotions (happiness, calmness) and lower levels of unpleasant post-race emotions (anger, confusion, depression, fatigue, tension).
Lane and Wilson, 2011	34 runners (8 females). Range = 23–59	SEIS	Marathon of Britain Race (175 miles, divided into 6 races)	BRUMS	Weak	TEI was associated with more pleasant emotions and less unpleasant emotions following the race.
Salminen and Ravaja, 2017	44 manager-subordinate dyads (18 female managers, 24 female subordinates). M (managers) = 43, (SD = 8.5. M (subordinates) = 41.9, SD = 9.0	SEIS	Performance review discussion	Self-assessment manikin	Weak	TEI was associated with more positive valence ratings for both managers and subordinates following the interaction.
Ruiz-Aranda et al., 2011	67 university students (57 females). *M* = 21.58, SD = .76	MSCEIT	Cold pressor task	Negative affect; affective pain (unpleasantness of stimulus); sensory pain (strength of stimulus)	Weak	AEI negatively correlated with NA, sensory pain, and affective pain.
Wilbraham et al., 2018	89 undergraduates in either stressful (n = 57, 42 female, *M* = 19.91, SD = 4.23) or control (*n* = 32, 27 female, *M* = 18.59, SD = 0.18) conditions	SUEIT	20 min oral presentation as part of coursework	Cortisol; Activation deactivation adjective checklist	Moderate	There were no main effects of TEI (or subscales) on cortisol levels or mood.

*MSCEIT, Mayer-Salovey-Caruso Emotional Intelligence Test; IAPS, International Affective Picture System; STAI-S, State Trait Anxiety Inventory—Short Form; POMS, Profile of Mood States; DSSQ, Dundee Stress State Questionnaire; NA, negative affect*.

##### Psychological reactivity

Higher TEI levels were strongly linked to more positive affect (and less negative affect) in sport-based stressors. A series of studies by Lane et al. showed that TEI promoted positive mood states during sports events, including a competition (Lane et al., 2009), a 10-mile running race (Lane et al., 2010), and a 175-mile marathon (Lane and Wilson, 2011). In each case, the high TEI participants had lower levels of negative emotions (e.g., anger, tension), and higher levels of positive emotions (e.g., happiness, calmness), a pattern associated with optimum performance (Lane et al., 2009). Higher employee TEI was also associated with a greater likelihood of experiencing positive emotions following a performance review discussion with a manager (Salminen and Ravaja, 2017).

Three studies examined the role of EI when responding to a painful stimulus. Two of those examined reactivity within a dental setting. During a dental procedure, children with higher TEI were less likely to display negative behavioral responses (e.g., crying, sudden body movements), than low TEI children (Aminabadi et al., 2011, 2013). The other study found that higher levels of AEI predicted less self-reported negative affect and pain during a cold pressor task, where the participant immerses their hand in freezing cold water (Ruiz-Aranda et al., 2011).

##### Physiological or mixed reactivity

As with psychological reactivity, findings were promising regarding TEI and physiological reactivity in a sporting context. During a pressurized sports activity, near-professional tennis players secreted less cortisol if they had higher TEI (Laborde et al., 2014). The same research group found similar findings with a different approach. Handball players were exposed to an auditory stressor that included negative sports-related sounds, such as crowds hissing (Laborde et al., 2011). When listening, the high TEI athletes experienced less cardiac reactivity compared to their low TEI counterparts.

TEI was less effective in other naturalistic settings. During an assessed presentation as part of an undergraduate psychology course, TEI neither increased nor decreased participants' cortisol levels (Wilbraham et al., 2018). Arora et al. (2011) focused on the capacity of TEI to buffer situational stress for medical students performing unfamiliar surgical tasks. While TEI was unrelated to HR reactivity, higher TEI was associated with increased psychological stress.

#### 5. Stress Recovery

A small number of studies (*n* = 6) included some assessment of stress recovery. In four of those cases, high EI individuals recovered faster than low EI individuals. For example, despite showing greater reactivity initially, high TEI individuals showed faster psychological recovery 15 min after watching an anger-provoking video (Fernández-Berrocal and Extremera, 2006), and after completing an unfamiliar task (Arora et al., 2011). However, Thomas et al. (2018) found no link between TEI and recovery 7 min after the group version of the TSST. TEI was related to *stronger* feelings of regret and disappointment 5 days after a failed negotiation (Sevdalis et al., 2007), a recovery period considerably longer than that used in the other studies. TEI was associated with stressor *habituation* (reduced reactivity upon extended/repeated exposure). Female university students that scored high on the emotional regulation TEI scale were less reactive when re-watching a distressing video depicting sexual assault that they had seen 2 days previously (Ramos et al., 2007). Another TEI scale—attention to emotions—also promoted habituation to the TSST (Salovey et al., 2002, Study 2).

AEI facilitated stress recovery in two studies. Limonero et al. (2015), assessed mood 15 min after exposure to emotional images. Mood returned to baseline faster for participants with higher scores on facilitation and understanding branches. Similarly, after recalling a sad memory, mood repair was faster when individuals had higher scores on the perception branch (Rash and Prkachin, 2013).

## Discussion

The final review identified 45 studies from 14 countries, from diverse settings including healthcare (e.g., Arora et al., 2011), sport (e.g., Lane and Wilson, 2011), organizational psychology (e.g., Salminen and Ravaja, 2017), and education (e.g., Wilbraham et al., 2018). This highlights that EI has cross-cultural and cross-disciplinary pertinence. The discussion section will (1) summarize the main findings, (2) discuss the measurement of EI across the studies reviewed, (3) identify study limitations, (4) discuss the limitations of this review, and, (5) suggest implications for EI in terms of adaptation, and propose future research directions.

### Summary of Main Findings

The first aim of the review was to examine the relationship between TEI, AEI, and stress reactivity and recovery. If EI is truly adaptive in acutely stressful conditions, high EI scorers should show the adaptive stress responding profile (i.e., reduced reactivity, faster recovery; Keefer et al., 2018). As expected, findings differed according to the EI type and stressor used.

#### Stress Reactivity: The Role of TEI

Overall, evidence concerning the role of TEI in psychological or physiological reactivity was mixed. Depending on the context, TEI increased reactivity, decreased reactivity, or had no significant effects. TEI appeared especially useful in sport. High TEI buffered reactivity to both passive (e.g., crowd hissing) and active (e.g., competition) sports-based stressors, a finding that was applicable to both psychological (e.g., Lane et al., 2010) and physiological stress (Laborde et al., 2014). The pertinence of TEI to sports-based stressors may reside in the structural basis of the construct. TEI can be conceptualized as “emotional self-efficacy”: one's self-confidence and belief in their emotional abilities (Petrides et al., 2007). Self-efficacy is one of the most influential determinants of sport performance, (Feltz et al., 2008), a phenomena that could be attributable to the many “rewards” available for performing well in sports contexts (e.g., winning a competition, beating a personal best, etc.). Incentives are deemed necessary for the “activation” of self-efficacy (Bandura, 1977). One could speculate that, as a related construct, TEI could work similarly by actively dampening the stress response in situations where “doing well” greatly benefits the individual (e.g., a marathon). Similarly, high TEI buffered affective responses in other “at risk” naturalistic settings where the individual was at risk of pain or physical discomfort.

TEI was unrelated to physiological responding when completing cognitive tasks under controlled conditions. However, the intensity of *affective* responses was buffered by TEI in most cases. Perhaps, during times of cognitive challenge, TEI facilitates deployment of adaptive cognitive mechanisms to regulate emotional responses. There has been relatively little evidence in the context of state coping (i.e., coping during the stressor). However, the limited body of work suggests that TEI facilitates coping strategy selection under acute stress (Salovey et al., 2002; Matthews et al., 2006; O'Connor et al., 2016). High TEI individuals typically select more adaptive, active methods of coping (e.g., problem-solving) over maladaptive, passive methods (e.g., avoidance coping; Austin et al., 2010). Furthermore, high TEI individuals appraise tasks as a challenge, rather than a threat (Mikolajczak and Luminet, 2008). This cognitive appraisal pattern fosters adaptive levels of reactivity, and enhances task performance (Maier et al., 2003). TEI is also associated with an attentional bias for positive emotions (Szczygieł and Mikolajczak, 2017; Lea et al., 2018), which could be helpful during demanding situations. For example: during a written exam, a student with greater TEI may experience less negative affect, allowing them to invest more mental resources in answering the exam questions, thus potentially resulting in greater academic achievement than a student with low TEI. What is less clear, is why high TEI did not protect individuals from socially evaluative stressors. TEI only reduced cortisol and mood reactivity in one study (Mikolajczak et al., 2009, study 3). In other studies, TEI or its component subscales either had no effect, or increased reactivity. Notably, when students delivered a presentation as part of their coursework (i.e., in a naturalistic setting), TEI failed to produce any effects on mood or cortisol reactivity (Wilbraham et al., 2018). Considering that enhanced emotional and social functioning should constitute a core hallmark of TEI (Fiori, 2009), findings challenge the claim that TEI buffers stress in all social contexts.

Many studies showed that TEI intensified emotional reactivity to material designed to evoke negative emotion (e.g., Petrides and Furnham, 2003, study 2). This could suggest that compared to their low TEI peers, high TEI individuals are more likely to notice their negative emotions and pay attention to them. Alternatively, rather than being the result of maladaptive psychological processing of the stressor, it could be that on those occasions, high TEI individuals believed they *should* be impacted negatively by negatively valenced material. They could have then over-reported this via subjective reports of mood change. Evidence exploring TEI and physiological reactions (free from demand bias) supports that hypothesis, since high TEI individuals did not necessarily show adaptive physiological responses to emotive material. However, the balance between TEI facets appeared important. For maximum benefit, individuals needed to score highly on their perceived ability to both perceive and regulate emotion.

#### Stress Reactivity: The Role of AEI

A dearth of AEI studies was apparent across all stressor types. However, based on the pool of evidence available within the review, findings were much less supportive of a role for AEI than TEI. AEI was either non-significant or detrimental in most cases. Notably, AEI was related to maladaptive physiological responses in intra-personal settings (e.g., Bechtoldt and Schneider, 2016). This contradicts suggestions that AEI should strongly predict adaptive criteria in such environments (Matthews et al., 2017). AEI also failed to predict reactivity to cognitive tasks (e.g., Matthews et al., 2006), and when confronted with emotive stimuli, findings were conflicted. In general, explanatory pathways with regard to AEI are less straight-forward, and it is difficult to speculate how and why AEI might implicate (or not implicate) the stress response pathway. It has been suggested by Ciarrochi et al. (2002) that maladaptive effects of AEI could stem from one of two possible accounts, where emotion perception skill plays a key role. First, emotionally perceptive people might be *hypersensitive* to emotion, and therefore less likely to try and repress the mental and physical sensations associated with negative experiences. Second, highly perceptive individuals might be *less confused* about what they are feeling, and are thus more aware of the meaning of such sensations. Taken together, findings align with contemporary concerns that high levels of AEI may not always be optimal for adaptation (Davis and Nichols, 2018).

The roles of both TEI and AEI in facilitating outcomes (i.e., stress reactivity) need to be understood (Davis and Humphrey, 2012). However, the vast majority of studies in the review explored the effects of TEI only, and only three studies examined *both* TEI and AEI simultaneously. Zysberg (2012) identified different roles for TEI and AEI (TEI; buffers psychological reactivity; AEI buffers physiological reactivity). The other two studies only examined effects on psychological reactivity. While both identified no benefit for high AEI (Fallon et al., 2014; Davis, 2018), TEI helped maintain positive mood in one case (Fallon et al., 2014). Even when studies used the same stress induction paradigm (TSST), and measurement (cortisol secretion), divergent findings were identified for TEI (less reactive; Mikolajczak et al., 2009) and AEI (more reactive; Bechtoldt and Schneider, 2016). This suggests that TEI and AEI may operate differently in stressor-activated processes. However, more studies evaluating respective roles of both TEI and AEI in stressful situations are clearly needed. Considering TEI/AEI “profiles” (high TEI/low AEI, high AEI/low TEI etc.), could prove a fruitful approach for future studies to take. It could be that the effects of AEI on stress reactivity (which were often negative or non-significant in the present review) depend on the level of TEI. For example, having high levels of emotional skill (AEI) can be deleterious for psychological adaptation if the individual does not possess a sufficient level of emotional self-confidence (TEI) (Davis and Humphrey, 2014).

#### Stress Recovery: The Roles of TEI and AEI

Recovery from acute stress is sometimes viewed an empirically neglected “conceptual sibling” of reactivity (Linden et al., 1997). A capacity to recover quickly from stress generally affords long term health benefits, by preventing exaggerated or prolonged activation of the sympathetic and HPA axis response systems (e.g., Burke et al., 2005; Geurts and Sonnentag, 2006). Few studies examined the role of EI in the stress recovery process. However, both TEI and AEI generally conveyed advantages for a range of stressful experiences. The mechanisms linking TEI and AEI to enhanced recovery are unknown, but the wider literature provides nascent support for the role of two related cognitive processes: post-stressor rumination (dwelling on the negative experience of the stressor after its end), and post-stressor intrusive thoughts (involuntary, unwelcome thoughts or images about the stressful experience). Lanciano et al. (2010) found that individuals that scored highly on the emotion management branch of AEI ruminated less about their stressful experiences. Similarly, people with high TEI (clarity of emotions subscale) experienced less intrusive thoughts (e.g., “I thought about [the stressor] when I didn't mean to”) post-stressor (Fernández-Berrocal and Extremera, 2006). Since rumination and intrusive thoughts can hinder the stress recovery process (LeMoult et al., 2013), it could follow that TEI and/or AEI might inhibit the focus on one's distress after the immediate threat has passed. Perhaps, via increased attendance to positive emotions (Szczygieł and Mikolajczak, 2017; Lea et al., 2018). More studies examining both TEI and AEI, using shared methodology, are required before conclusions about their roles with respect to acute stress recovery can be confidently drawn.

### Measurement of TEI and AEI

A second aim of the review concerned the typical methodology (e.g., EI instrumentation) used when exploring the effects of EI on acute stress responding. A considerable problem in the field of EI is that there is no clear definition or “gold standard” measures. This has resulted in a plethora of measures, particularly for TEI, which differ in their theoretical assumptions and factor structures (Zeidner and Matthews, 2018). For example, unlike other popular TEI measures such as the TEIQue, the TMMS does not yield a global score, and lacks many core facets of the TEI construct, such as sociability (Pérez et al., 2005). Thus, synthesizing findings that relate to different TEI conceptualisations may not be valid. Eventually, with more studies, and replication of methods, a meta-analysis could determine strength of effects according to EI instrumentation and stressor type. Studies also differed in their analytic strategy. Heterogeneity of methodology means that at present, testing for a “common effect” in this way would not be possible. While half of the studies only performed analyses at the global level (i.e., total score), the rest followed a promising line of enquiry by performing sub-analyses with EI components, which helps to pinpoint effects at the sub-facet level. In those studies, significant effects were often restricted to certain subscales (e.g., clarity scale of the TMMS; Fernández-Berrocal and Extremera, 2006), supporting that strategy. In addition, subscale analysis would help address the extent to which certain EI subscales (e.g., the wellbeing scale of the TEIQue) confound with stress outcomes. What is problematic, however, is when studies only measured/reported select subscales from a broader measure (e.g., Papousek et al., 2008, 2011), as this makes it more difficult to elucidate EI's role.

A large number of studies examined the relationship between TEI (i.e., *self-reported* EI) and psychological reactivity (i.e., *self-reported* stress). When both predictors and criterion measures are self-reported, there is the risk that findings may have arisen due to shared measurement error, rather than true associations (“contamination”; Keefer et al., 2018). Thus, the effects of TEI on health indices tend to be weaker when outcomes are measured objectively, as shown in the present review. In addition, self-report behavioral trait questionnaires assume individuals have sufficient insight into their own emotional functioning, and are thus susceptible to socially desirable responding (Day and Carroll, 2008; Tett et al., 2012). It is therefore important to consider TEI findings alongside those for AEI, a more objective index of emotional skills and abilities. However, as discussed, few studies examined AEI. In those few studies, a narrow breadth were used, with the majority of studies using the MSCEIT. Commentators argue that implementation of alternative measurement tools is required to fully differentiate test effects from construct effects and avoid “mono-method bias” (Matthews et al., 2007). In other words, researchers should use a range of AEI tools to demonstrate that effects are not merely a product of the way in which the MSCEIT measures emotional skills. Non-commercial alternatives have since been developed to address this need (e.g., STEM and STEU; MacCann and Roberts, 2008), though these are not often used, as reflected by present review (see Table 2).

### Study Limitations

The quality appraisal process showed that of the 45 studies, most conferred a weak (*n* = 18) or moderate (*n* = 21) rating. A strong rating was only received by four studies (see Tables 3–6). The main issues—the dearth of evidence for physiological reactivity studies, stress induction robustness, and, lack of consideration for confounding influences—will now be discussed.

Only a third of studies assessed physiological stress. This is congruent with the findings relating to EI measurement: researchers in the review tended to select subjective measures (i.e., TEI) over objective measures (i.e., AEI). Assessment of physiology in reactivity experiments could prove particularly insightful, given that the physiological aspects of reactivity are strongly associated with adverse health outcomes (e.g., Lopez-Duran et al., 2015). Using physiological measures also reduces the risk of methodological “contamination” occurring from an overreliance on self-report (described above). Furthermore, we cannot assume that perceived stress adequately represents physiology, since the literature often indicates negligible associations (Oldehinkel et al., 2011). Indeed, one meta-analysis concluded that significant correlations between perceived stress and physiological stress are only found in approximately 25% of cases (Campbell and Ehlert, 2012). Of the few studies in the review that captured both types of stress measurement, effects were rarely consistent across both. The degree and strength of concordance can depend on many factors, such as age, gender, and body composition (Föhr et al., 2015). For those reasons, multi-method approaches (i.e., using physiological methods alongside questionnaires) are preferred (Andrews et al., 2013). Some also argue that to truly understand the full body response, both ANS (e.g., HR) and HPA-axis (e.g., cortisol) markers should be measured, since these systems are highly coordinated and interconnected (Rotenberg and McGrath, 2016). Future work should continue to evaluate the respective roles of TEI and AEI in stressful situations using both psychological and physiological measurements.

Another key issue relates to the robustness of stress induction paradigms used. A broad range of stress induction procedures were identified in the review (see Figure 2). Only 10 studies (22%) included an explicit control group (i.e., high stress vs. low stress conditions). The remaining 34 studies had either no control group at all (*n* = 25), used intrasubject control (e.g., consecutive conditions; *n* = 5), or had multiple conditions (e.g., happy mood; sad mood) without a neutral condition (*n* = 5). Experimental control is a crucial component of the scientific method (Bowling, 2009) that reduces the risk of bias arising from environmental influences. Moreover, two thirds of the studies did not control for any additional variables that might have confounded with EI to influence reactivity or recovery variables, such as personality, cognitive ability, or mental health. Considering TEI is widely acknowledged as a lower order personality trait (Petrides et al., 2007), it is concerning that TEI studies do not routinely account for personality. Similarly, only two AEI studies controlled for cognitive ability, a closely linked construct to AEI (Mayer et al., 2008). Acute stress responding can also be influenced by clinical symptomology. For example, individuals with depression (Burke et al., 2005) or anxiety (De Rooij et al., 2010), often show blunted stress reactivity, and impaired stress recovery, compared to controls. Levels of trait anxiety and depression were only accounted for in one study (Mikolajczak et al., 2009, study 2). It is difficult to clearly define the relationship between EI and stress responding when the effects of confounding influences are not controlled for. Although the incremental validity of EI in a wide range of criteria is promising (Andrei et al., 2016; Miao et al., 2018), to further establish the contribution of EI toward outcomes, researchers should aim to include measurement of emotion-related constructs in EI studies. Differences in methodological robustness could help to explain conflicting findings identified in the review. For example, Mikolajczak et al. (2009, study 3, which identified decreased reactivity) and Thomas et al. (2018, which identified increased reactivity), used variants of the same stressful task (TSST), the same TEI measure (TEIQue), and stress measurement (cortisol secretion). However, unlike the latter study, the former employed a control group, and controlled for confounding variables.

### Limitations of the Review

At the review level, publication bias emerged. Two unpublished theses of potential relevance could not be obtained despite attempts to contact the authors.

### Conclusions and Future Directions

Over the last two decades, EI has been claimed to hold a pivotal role with regards to many intrapersonal and interpersonal adaptive life outcomes. A key hypothesis suggests that EI leads to those positive outcomes by acting as an acute stress buffer. The present systematic review provides a timely overview of the experimental literature concerning EI and acute stress reactivity and recovery, bringing together relevant work from a vast array of disciplines. The hypothesis was only partially supported by the results of the present review. Findings suggested that whether EI is useful under acute stress is highly dependent on the stress context, and how EI is measured. TEI was significantly associated with reduced stress reactivity in two contexts: sports-based stressors (e.g., a sports competition), and cognitive stressors (e.g., a memory task), but not others (psychosocial stress; emotive stimuli). Furthermore, relationships between EI and self-reported stress generally occurred more often than with physiological stress (a more reliable index of reactivity). It was also unclear whether AEI, a more objective index of emotional skill, was adaptive, since relatively few studies measured this construct, and some indicated a *deleterious* effect of AEI. However, while emotionally intelligent individuals may or may not react more strongly to a stressor, they do seem to recover more quickly from the ordeal, regardless of how EI or stress is measured.

The review also identified some core limitations, which researchers should attempt to address in future studies. First, research concerning EI and reactivity should strive for experimental rigor. While some high quality studies (e.g., Mikolajczak et al., 2009, study 3) used effective stress manipulations (with appropriate controls), controlled for confounding constructs, and considered multiple indices of reactivity, these were scarce. Second, it would be beneficial for the field for more studies to examine the contribution of both actual emotional skills (AEI) in addition to trait emotional self-efficacy (TEI). Importantly, it is also not possible to generalize findings to other populations (e.g., adolescents), given that most study samples were restricted to University students. Considering the drive to train or improve EI in children and young people, a third recommendation would be for future studies to examine the relationship between EI and stress reactivity in those populations. Alternatively, a novel approach would be to utilize virtual reality technology, exploring the role of EI when responding to a wide range of naturalistic stimuli and scenarios, without the practical restraints of current laboratory-based research. Overall, the findings of the review call into question some central assumptions about the stress-buffering effect of EI, and suggest that EI may only be useful in certain circumstances.

## Author Contributions

RL was the primary researcher of this study, responsible for collecting and analyzing the data, and writing the first draft of the paper. SD was responsible for analyzing data and editing the paper. BM and PQ were also responsible for editing the paper. All authors contributed to the conceptualization of the review.

### Conflict of Interest Statement

The authors declare that the research was conducted in the absence of any commercial or financial relationships that could be construed as a potential conflict of interest.

## Footnotes

^1^Database searching revealed another paper of interest (Mikolajczak et al., 2007). However, the data from that paper was also reported in Mikolajczak et al. (2009) Study 3. Thus, the latter paper was included in lieu.

## References

[B1] ^*^ Agnoli S. Pittarello A. Hysenbelli D. Rubaltelli E. (2015). “Give, but Give until it Hurts”: The modulatory role of trait emotional intelligence on the motivation to help. PLoS ONE 10:e0130704 10.1371/journal.pone.0130704 26121350 PMC4487050

[B2] ^*^ Aminabadi N. Adhami Z. Oskouei S. G. Najafpour E. Jamali Z. (2013). Emotional intelligence subscales: are they correlated with child anxiety and behavior in the dental setting? J. Clin. Pediatr. Dentistr. 38, 61–66. 10.17796/jcpd.38.1.k754h164m3210764 24579285

[B3] ^*^ Aminabadi N. Erfanparast L. Adhami Z. E. Maljaii E. Ranjbar F. Jamali Z. (2011). The impact of emotional intelligence and intelligence quotient (IQ) on child anxiety and behaviour in the dental setting. Acta Odontol. Scand. 69, 292–298. 10.3109/00016357.2011.568959 21426272

[B4] Andrei F. Siegling A. B. Aloe A. M. Baldaro B. Petrides K. V. (2016). The incremental validity of the Trait Emotional Intelligence Questionnaire (TEIQue): a systematic review and meta-analysis. J. Pers. Assess. 98, 261–276. 10.1080/00223891.2015.1084630 26457443

[B5] Andrews J. Ali N. Pruessner J. C. (2013). Reflections on the interaction of psychogenic stress systems in humans: the stress coherence/compensation model. Psychoneuroendocrinology 38, 947–961. 10.1016/j.psyneuen.2013.02.010 23522990

[B6] Armijo-Olivo S. Stiles C. R. Hagen N. A. Biondo P. D. Cummings G. G. (2012). Assessment of study quality for systematic reviews: a comparison of the cochrane collaboration risk of bias tool and the effective public health practice project quality assessment tool: methodological research. J. Eval. Clin. Pract. 18, 12–18. 10.1111/j.1365-2753.2010.01516.x 20698919

[B7] ^*^ Arora S. Russ S. Petrides K. V. Sirimanna P. Aggarwal R. Sevdalis N. (2011). Emotional intelligence and stress in medical students performing surgical tasks. Acad. Med. 86, 1311–1317. 10.1097/ACM.0b013e31822bd7aa 21869667

[B8] Arora S. Sevdalis N. Nestel D. Woloshynowych M. Darzi A. Kneebone R. (2010). The impact of stress on surgical performance: a systematic review of the literature. Surgery 147, 318–330. 10.1016/j.surg.2009.10.007 20004924

[B9] Austin E. J. Saklofske D. H. Mastoras S. M. (2010). Emotional intelligence, coping and exam-related stress in Canadian undergraduate students. Aust. J. Psychol. 62, 42–50. 10.1080/00049530903312899

[B10] Bandura A. (1977). Self-effiacy: toward a unifying theory of behavioural change. Psychol. Rev. 84, 191–215. 10.1037/0033-295X.84.2.191 847061

[B11] Bar-On R. Parker J. D. A. (2000). The Bar-On Emotional Quotient Inventory: Youth Version (EQ-i:YV) Technical Manual. Toronto, ON: Multi-Health Systems, Inc.

[B12] Baumann N. Turpin J. C. (2010). Neurochemistry of stress. an overview. Neurochem. Res. 35, 1875–1879. 10.1007/s11064-010-0298-9 20978849

[B13] ^*^ Bechtoldt M. N. Schneider V. K. (2016). Predicting stress from the ability to eavesdrop on feelings: emotional intelligence and testosterone jointly predict cortisol reactivity. Emotion 16, 815–825. 10.1037/emo0000134 27064289

[B14] Bowling A. (2009). Research Methods in Health: Investigating Health and Health Services. Berkshire: Open University Press.

[B15] Brackett M. Rivers M. Salovey P. (2011). Emotional intelligence: Implications for personal, social, academic, and workplace success. Soc. Personal. Psychol. Compass 5, 88–103. 10.1111/j.1751-9004.2010.00334.x

[B16] Brackett M. A. Rivers S. E. Shiffman S. Lerner N. Salovey P. (2006). Relating emotional abilities to social functioning: a comparison of self-report and performance measures of emotional intelligence. J. Pers. Soc. Psychol. 91, 780–795. 10.1037/0022-3514.91.4.780 17014299

[B17] Brannick M. T. Wahi M. M. Arce M. Johnson H. A. Nazian S. Goldin S. B. (2009). Comparison of trait and ability measures of emotional intelligence in medical students. Med. Educ. 43, 1062–1068. 10.1111/j.1365-2923.2009.03430.x 19874499

[B18] Burke H. M. Davis M. C. Otte C. Mohr D. C. (2005). Depression and cortisol responses to psychological stress: a meta-analysis. Psychoneuroendocrinology 30, 846–856. 10.1016/j.psyneuen.2005.02.010 15961250

[B19] Campbell J. Ehlert U. (2012). Acute psychosocial stress: Does the emotional stress response correspond with physiological responses? Psychoneuroendocrinology 37, 1111–1134. 10.1016/j.psyneuen.2011.12.010 22260938

[B20] Cha C. Nock M. (2009). Emotional intelligence is a protective factor for suicidal behaviour. J. Am. Acad. Child Adolesc. Psychiatry 48, 422–430. 10.1097/CHI.0b013e3181984f44 19318882

[B21] Chida Y. Hamer M. (2008). Chronic psychosocial factors and acute physiological responses to laboratory-induced stress in healthy populations: a quantitative review of 30 years of investigations. Psychol. Bull. 134, 829–885. 10.1037/a0013342 18954159

[B22] Chida Y. Steptoe A. (2010). Greater cardiovascular responses to laboratory mental stress are associated with poor subsequent cardiovascular risk status: a meta-analysis of prospective evidence. Hypertension 55, 1026–1032. 10.1161/HYPERTENSIONAHA.109.146621 20194301

[B23] Ciarrochi J. Deane F. P. Anderson S. (2002). Emotional intelligence moderates the relationship between stress and mental health. Pers. Individ. Dif. 32, 197–209. 10.1016/S0191-8869(01)00012-5

[B24] ^*^ Ciarrochi J. V. Chan A. Y. C. Bajgar J. (2001). Measuring emotional intelligence in adolescents. Pers. Individ. Dif. 31, 1105–1119. 10.1016/S0191-8869(00)00207-5

[B25] ^*^ Davis S. K. (2018). Emotional intelligence and attentional bias for threat-related emotion under stress. Scand. J. Psychol. 59, 328–339. 10.1111/sjop.12439 29569275

[B26] Davis S. K. Humphrey N. (2012). The influence of emotional intelligence (EI) on coping and mental health in adolescence: divergent roles for trait and ability EI. J. Adolesc. 35, 1369–1379. 10.1016/j.adolescence.2012.05.007 22704492

[B27] Davis S. K. Humphrey N. (2014). Ability versus trait emotional intelligence. J. Indiv. Dif. 35, 54–62. 10.1027/1614-0001/a000127

[B28] Davis S. K. Nichols R. (2018). Does emotional intelligence have a “dark” side? A review of the literature. Front. Psychol. 7:1316. 10.3389/fpsyg.2016.01316 27625627 PMC5003940

[B29] Day A. L. Carroll S. A. (2008). Faking emotional intelligence (EI): comparing response distortion on ability and trait-based EI measures. J. Organ. Behav. 29, 761–784. 10.1002/job.485

[B30] Day A. L. Therrien D. L. Carroll S. A. (2005). Predicting psychological health: assessing the incremental validity of emotional intelligence beyond personality, type a behaviour, and daily hassles. Eur. J. Pers. 19, 519–536. 10.1002/per.552

[B31] De Rooij S. R. Schene A. H. Phillips D. I. Roseboom T. J. (2010). Depression and anxiety: associations with biological and perceived stress reactivity to a psychosocial stress protocol in a middle-aged population. Psychoneuroendocrinology 35, 866–877. 10.1016/j.psyneuen.2009.11.011 20031333

[B32] Denson T. F. Spanovic M. Miller N. (2009). Cognitive appraisals and emotions predict cortisol and immune responses: a meta-analysis of acute laboratory social stressors and emotion inductions. Psychol. Bull. 135, 823–853. 10.1037/a0016909 19883137

[B33] Effective Public Health Practice Project (EPHPP) (1998). Quality Assessment Tool for Quantitative Studies. Available online at: https://merst.ca/ephpp/ (accessed December 1, 2018).

[B34] Extremera N. Durán A. Rey L. (2007). Perceived emotional intelligence and dispositional optimism-pessimism: analyzing their role in predicting psychological adjustment among adolescents. Pers. Individ. Dif. 42, 1069–1079. 10.1016/j.paid.2006.09.014

[B35] ^*^ Fallon C. K. Panganiban A. R. Wohleber R. Matthews G. Kustubayeva A. M. Roberts R. (2014). Emotional intelligence, cognitive ability and information search in tactical decision making. Pers. Individ. Dif. 65, 24–29. 10.1016/j.paid.2014.01.029

[B36] ^*^ Fellner A. N. Matthews G. Shockley K. D. Warm J. S. Zeidner M. Karlov L. (2012). Using emotional cues in a discrimination learning task: effects of trait emotional intelligence and affective state. J. Res. Pers. 46, 239–247. 10.1016/j.jrp.2012.01.004

[B37] Feltz D. L. Short S. E. Sullivan P. J. (2008). Self-Efficacy in Sport. Champaign, IL: Human Kinetics.

[B38] ^*^ Fernández-Berrocal P. Extremera N. (2006). Emotional intelligence and emotional reactivity and recovery in laboratory context. Psiotherma 18, 72–78. 17295961

[B39] Fiori M. (2009). A new look at emotional intelligence: a dual-process framework. Pers. Soc. Psychol. Rev. 13, 21–44. 10.1177/1088868308326909 19114503

[B40] Föhr T. Talvonen A. Myllymäki T. Järvelä-Reijonen E. Rantala S. Korpela R.. (2015). Subjective stress, objective heart rate variability-based stress, and recovery on workdays among overweight and psychologically distressed individuals: a cross-sectional study. J. Occup. Med. Toxicol. 10:39. 10.1186/s12995-015-0081-6 26504485 PMC4620623

[B41] Freudenthaler H. H. Neubauer A. C. (2005). Emotional intelligence: the convergent and discriminant validities of intra- and interpersonal emotional abilities. Pers. Individ. Dif. 39, 569–579. 10.1016/j.paid.2005.02.004

[B42] Geurts S. A. Sonnentag S. (2006). Recovery as an explanatory mechanism in the relation between acute stress reactions and chronic health impairment. Scand. J. Work Environ. Health 32, 482–492. 10.5271/sjweh.1053 17173204

[B43] ^*^ Gohm C. L. (2003). Mood regulation and emotional intelligence: individual differences. J. Pers. Soc. Psychol. 84, 594–607. 10.1037/0022-3514.84.3.594 12635919

[B44] Harms P. D. Credé M. (2010). Emotional intelligence and transformational and transactional leadership: a meta-analysis. J. Leadership Organ. Stud. 17, 5–17. 10.1177/1548051809350894

[B45] Henze G. I. Zänkert S. Urschler D. F. Hiltl T. J. Kudielka B. M. Pruessner J. C.. (2017). Testing the ecological validity of the Trier Social Stress Test: association with real-life exam stress. Psychoneuroendocrinology 75, 52–55. 10.1016/j.psyneuen.2016.10.002 27771565

[B46] Heopniemi T. Elovainio M. Pulkki L. Puttonen S. Raitakari O. Keltikangas-Jarvinen L. (2007). Cardiac autonomic reactivity and recovery in predicting carotid atherosclerosis: the cardiovascular risk in young Finns study. Health Psychol. 26, 13–21. 10.1037/0278-6133.26.1.13 17209693

[B47] Hu M. Lamers F. de Geus E. Penninx B. (2016). Differential autonomic nervous system reactivity in depression and anxiety during stress depending on type of stressor. Psychosom. Med. 78, 562–572. 10.1097/PSY.0000000000000313 26910796

[B48] Keefer K. V. Saklofske D. H. Parker J. D. A. (2018). Emotional intelligence, stress, and health: when the going gets tough, the tough turns to emotions, in An Introduction to Emotional Intelligence. eds Dacre Pool L. Qualter P. (Sussex: Wiley), 161–184. 10.1007/978-3-319-90633-1

[B49] Kirschbaum C. Pirke K. M. Hellhammer D. H. (1993). The ‘Trier Social Stress Test': a tool for investigating psychobiological stress responses in a laboratory setting. Neuropsychobiology 28, 76–81. 8255414 10.1159/000119004

[B50] Krkovic K. Clamor A. Lincoln T. M. (2018). Emotion regulation as a predictor of the endocrine, autonomic, affective, and symptomatic stress response and recovery. Psychoneuroendocrinology 94, 112–120. 10.1016/j.psyneuen.2018.04.028 29775874

[B51] Kuhlmann S. Piel M. Wolf O. T. (2005). Impaired memory retrieval after psychosocial stress in healthy young men. J. Neurosci. 25, 2977–2982. 10.1523/JNEUROSCI.5139-04.2005 15772357 PMC6725125

[B52] ^*^ Laborde S. Brüll A. Weber J. Anders L. S. (2011). Trait emotional intelligence in sports: a protective role against stress through heart rate variability? Pers. Individ. Dif. 51, 23–27. 10.1016/j.paid.2011.03.003

[B53] ^*^ Laborde S. Dosseville F. Scelles N. (2010). Trait emotional intelligence and preference for intuition and deliberation: respective influence on academic performance. Pers. Individ. Dif. 49, 784–788. 10.1016/j.paid.2010.06.031

[B54] ^*^ Laborde S. Lautenbach F. Allen M. S. Herbert C. Achtzehn S. (2014). The role of trait emotional intelligence in emotion regulation and performance under pressure. Pers. Individ. Dif. 57, 43–47. 10.1016/j.paid.2013.09.013

[B55] Lanciano T. Curci A. Zatton E. (2010). Why do some people ruminate more or less than others? The role of emotional intelligence ability. Eur. J. Psychol. 6, 65–84. 10.5964/ejop.v6i2.185

[B56] ^*^ Lane A. M. Devonport T. J. Soos I. Karsai I. Leibinger E. Hamar P. (2010). Emotional intelligence and emotions associated with optimal and dysfunctional athletic performance. J. Sports Sci. Med. 9, 388–392. 24149631 PMC3761705

[B57] ^*^ Lane A. M. Thelwell R. Devonport T. J. (2009). Emotional intelligence and mood states associated with optimal performance. E J. Appl. Psychol. 5, 67–73. 10.7790/ejap.v5i1.123

[B58] ^*^ Lane A. M. Wilson M. (2011). Emotions and trait emotional intelligence among ulta-endurance runners. J. Sci. Med. Sport 14, 358–362. 10.1016/j.jsams.2011.03.001 21440500

[B59] Lea R. G. Qualter P. Davis S. Pérez-González J. C. Bangee M. (2018). Trait emotional intelligence and attentional bias for positive emotion: an eye-tracking study. Pers. Individ. Dif. 128, 88–93. 10.1016/j.paid.2018.02.017

[B60] LeBlanc V. R. (2009). The effects of acute stress on performance: Implications for health professions education. Acad. Med. 84, 25–33. 10.1097/ACM.0b013e3181b37b8f 19907380

[B61] LeMoult J. Arditte K. A. D'Avanzato C. Joorman J. (2013). State rumination: associations with emotional stress reactivity and attention biases. J. Exp. Psychopathol. 4, 471–484. 10.5127/jep.029112 25431652 PMC4243309

[B62] ^*^ Limonero J. T. Fernández-Castro J. Soler-Oritja J. Álvarez-Moleiro M. (2015). Emotional intelligence and recovering from induced negative emotional state. Front. Psychol. 6:816. 10.3389/fpsyg.2015.00816 26150794 PMC4472988

[B63] Linden W. Earle T. L. Gerin W. Christenfeld N. (1997). Physiological stress reactivity and recovery: conceptual siblings separated at birth? J. Psychosom. Res. 42, 117–135. 10.1016/S0022-3999(96)00240-1 9076640

[B64] ^*^ Ling S. Raine A. Gao Y. Schug R. (2018). The mediating role of emotional intelligence on the autonomic functioning - psychopathy relationship. Biol. Psychol. 136, 136–143. 10.1016/j.biopsycho.2018.05.012 29879434

[B65] Lopez-Duran N. L. McGinnis E. Kuhlman K. Geiss E. Vargas I. Mayer S. (2015). HPA-axis stress reactivity in youth depression: evidence of impaired regulatory processes in depressed boys. Stress 15, 545–553. 10.3109/10253890.2015.1053455 PMC5403248 26115161

[B66] MacCann C. Roberts R. D. (2008). New paradigms for assessing emotional intelligence: theory and data. Emotion 8, 540–551. 10.1037/a0012746 18729584

[B67] Maier K. J. Waldstein S. R. Synowski S. J. (2003). Relation of cognitive appraisal to cardiovascular reactivity, affect, and task engagement. Ann. Behav. Med. 26, 32–41. 10.1207/S15324796ABM2601_05 12867352

[B68] Martins A. Ramalho N. C. Morin E. M. (2010). A comprehensive meta-analysis of the relationship between emotional intelligence and health. Pers. Individ. Dif. 49, 554–564. 10.1016/j.paid.2010.05.029

[B69] ^*^ Matthews G. Emo A. K. Funke G. Zeidner M. Roberts R. Costa P. T.. (2006). Emotional intelligence, personality and task-induced stress. J. Exp. Psychol. Appl. 12, 96–107. 10.1037/1076-898X.12.2.96 16802891

[B70] ^*^ Matthews G. Pérez-González J. C. Fellner A. N. Funke G. Emo A. K. Zeidner M. (2015). Individual differences in facial emotion processing: trait emotional intelligence, cognitive ability, or transient stress? J. Psychoeduc. Assess. 33, 68–82. 10.1177/0734282914550386

[B71] Matthews G. Zeidner M. Roberts R. (2017). Emotional intelligence, health, and stress, in The Handbook of Stress and Health: A Guide to Research and Practice. eds Cooper C. L. Quick J. C. (Sussex: Wiley), 312–326. 10.1002/9781118993811.ch18

[B72] Matthews G. Zeidner M. Roberts R. D. (2007). Measuring emotional intelligence: promises, pitfalls, solutions?, in Handbook of Methods in Positive Psychology. eds Ong A. D. Van Dulmen M. (Oxford: Oxford University Press), 189–204.

[B73] Matthews K. A. Katholi C. R. McCreath H. Whooley M. A. Williams D. R. Zhu S.. (2004). Blood pressure reactivity to psychological stress predicts hypertension in the CARDIA study. Circulation 110, 74–78. 10.1161/01.CIR.0000133415.37578.E4 15210592

[B74] Mayer J. Roberts R. Barsade S. (2008). Human abilities: emotional intelligence. Annu. Rev. Psychol. 59, 507–536. 10.1146/annurev.psych.59.103006.093646 17937602

[B75] Mayer J. D. Salovey P. Caruso D. R. (2002). Mayer-Salovey-Caruso Emotional Intelligence Test (MSCEIT) User's Manual. Toronto, ON: Multi-Health Systems.

[B76] McEwen B. S. (2006). Stress, adaptation and disease: allostasis and allostatic load. Ann. N. Y. Acad. Sci. 840, 33–44. 10.1111/j.1749-6632.2001.tb05830.x 9629234

[B77] McEwen B. S. (2017). Neurobiological and systemic effects of chronic stress. Chronic Stress 1. 10.1177/2470547017692328 28856337 PMC5573220

[B78] Miao C. Humphrey R. Shanshan Q. (2018). A cross-cultural meta-analysis of how leader emotional intelligence influences subordinate task performance and organizational citizenship behavior. J. World Business 53, 463–474. 10.1016/j.jwb.2018.01.003

[B79] Mikolajczak M. Luminet O. (2008). Trait emotional intelligence and the cognitive appraisal of stressful events: an exploratory study. Pers. Individ. Dif. 44, 1445–1453. 10.1016/j.paid.2007.12.012

[B80] Mikolajczak M. Luminet O. Menil C. (2006). Predicting resistance to stress: incremental validity of trait emotional intelligence over alexithymia and optimism. Psiotherma 18, 79–88. 17295962

[B81] ^*^ Mikolajczak M. Petrides K. V. Coumans N. Luminet O. (2009). The moderating effect of trait emotional intelligence on mood deterioration following laboratory-induced stress. Int. J. Clin. Health Psychol. 9, 455–477. Available online at: https://psycnet.apa.org/record/2009-16945-007

[B82] Mikolajczak M. Roy E. Luminet O. Fillée C. de Timary P. (2007). The moderating impact of emotional intelligence on free cortisol responses to stress. Psychoneuroendocrinology 32, 1000–1012. 10.1016/j.psyneuen.2007.07.009 17935898

[B83] Moher D. Liberati A. Tetzlaff J. Altman D. G. (2009). Preferred reporting items for systematic reviews and meta-analyses: the PRISMA statement. Ann. Inter. Med. 151, 264–269. 10.7326/0003-4819-151-4-200908180-00135 19622511

[B84] Nater U. M. Okere U. Stallkamp R. Moor C. Ehlert U. Kliegel M. (2006). Psychosocial stress enhances time-based prospective memory in healthy young men. Neurobiol. Learn. Mem. 86, 344–348. 10.1016/j.nlm.2006.04.006 16753313

[B85] Nelis D. Kotsou I. Quoidbach J. Hansenne M. Weytens F. Dupuis P.. (2011). Increasing emotional competence improves psychological and physical well-being, social relationships, and employability. Emotion 11, 354–366. 10.1037/a0021554 21500904

[B86] ^*^ O'Connor P. Nguyen J. Anglim J. (2016). Effectively coping with task stress: a study of the validity of the trait emotional intelligence questionnaire- short form (TEIQue-SF). J. Pers. Assess. 99, 304–314. 10.1080/00223891.2016.1226175 27690638

[B87] O'Donnell K. Brydon L. Wright C. E. Steptoe A. (2008). Self-esteem levels and cardiovascular and inflammatory responses to acute stress. Brain Behav. Immun. 22, 1241–1247. 10.1016/j.bbi.2008.06.012 18644432

[B88] Oldehinkel A. J. Ormel J. Bosch N. M. Bouma E. M. C. Van Roon A. M. Rosmalen J. G. M.. (2011). Stressed out? Associations between perceived and physiological stress responses in adolescents: the TRAILS study. Psychophysiology 48, 441–452. 10.1111/j.1469-8986.2010.01118.x 21361964

[B89] Palmer B. Walls M. Burgess Z. Stough C. (2001). Emotional intelligence and effective leadership. Leadership Organ. Dev. J. 22, 5–10. 10.1108/01437730110380174

[B90] Panagi L. Poole L. Hackett R. A. Steptoe A. (2018). Happiness and inflammatory responses to acute stress in people with Type 2 diabetes. Ann. Behav. Med. 53, 309–320. 10.1093/abm/kay039 29924291 PMC6426003

[B91] ^*^ Papousek I. Freudenthaler H. Schulter G. (2008). The interplay of perceiving and regulating emotions in becoming infected with positive and negative moods. Pers. Individ. Dif. 45, 463–467. 10.1016/j.paid.2008.05.021

[B92] ^*^ Papousek I. Freudenthaler H. Schulter G. (2011). Typical performance measures of emotion regulation and emotion perception and frontal EEG asymmetry in an emotional contagion paradigm. Pers. Individ. Dif. 51, 1018–1022. 10.1016/j.paid.2011.08.013

[B93] Pérez J. C. Petrides K. V. Furnham A. (2005). Measuring trait emotional intelligence, in Emotional Intelligence: An International Handbook. eds Schulze R. Roberts R. D. (Cambridge, MA: Hogrefe and Huber), 181–201.

[B94] Petrides K. Pita R. Kokkinaki F. (2007). The location of trait emotional intelligence in personality factor space. Br. J. Psychol. 98, 273–289. 10.1348/000712606X120618 17456273

[B95] Petrides K. V. (2009). Psychometric properties of the Trait Emotional Intelligence Questionnaire, in Advances in the Assessment of Emotional Intelligence. eds Stough C. Saklofske D. H. Parker J. D. (New York, NY: Springer), 21–33. 10.1007/978-0-387-88370-0_5

[B96] ^*^ Petrides K. V. Furnham A. (2003). Trait emotional intelligence: behavioural validation in two studies of emotion recognition and reactivity to mood induction. Eur. J. Pers. 17, 39–57. 10.1002/per.466

[B97] Petrides K. V. Mikolajczak M. Mavroveli S. Sanchez-Ruiz M. J. Furnham A. Pérez-González J. C. (2016). Developments in trait emotional intelligence research. Emotion Rev. 8, 335–341. 10.1177/1754073916650493

[B98] Phillips A. C. Ginty A. T. Hughes B. M. (2013). The other side of the coin: blunted cardiovasculat and cortisol reactivity are associated with negative health outcomes. Int. J. Psychophysiol. 90, 1–7. 10.1016/j.ijpsycho.2013.02.002 23454029

[B99] ^*^ Pittarello A. Conte B. Caserotti M. Scrimin S. Rubaltelli E. (2018). Emotional intelligence buffers the effect of physiological arousal on dishonesty. Psychol. Bull. Rev. 25, 440–446. 10.3758/s13423-017-1285-9 28409438 PMC5862927

[B100] ^*^ Ramos N. Fernández-Berrocal P. Extremera N. (2007). Perceived emotional intelligence facilitates cognitive-emotional processes of adaptation to an acute stressor. Cogn. Emotion 21, 758–772. 10.1080/02699930600845846

[B101] Rano J. Fridén C. Eek. F. (2018). Effects of acute psychological stress on athletic performance in elite male swimmers. J. Sports Med. Phys. Fitness. 10.23736/S0022-4707.18.08493-1. [Epub ahead of print]. 29845840

[B102] ^*^ Rash J. A. Prkachin K. M. (2013). Cardiac vagal reactivity during relived sadness is predicted by affect intensity and emotional intelligence. Biol. Psychol. 92, 106–113. 10.1016/j.biopsycho.2012.11.009 23182876

[B103] Roberts R. D. Burrus J. Betancourt A. C. Holtzman S. Libbrecht N. MacCann C. (2013). Multimedia Assessment of Emotional Abilities: Development and Validation. Princeton, NJ: Educational Testing Service.

[B104] Rotenberg S. McGrath J. (2016). Inter-relation between autonomic and HPA axis activity in children and adolescents. Biol. Psychol. 117, 16–25. 10.1016/j.biopsycho.2016.01.015 26835595 PMC5731846

[B105] Ruiz-Aranda D. Castillo R. Salguero J. M. Cabello R. Fernández-Berrocal P. Balluerka N. (2012). Short- and midterm effects of emotional intelligence training on adolescent mental health. J. Adoles. Health 51, 462–467. 10.1016/j.jadohealth.2012.02.003 23084167

[B106] ^*^ Ruiz-Aranda D. Salguero J. M. Fernández-Berrocal P. (2011). Emotional intelligence and acute pain: the mediating effect of negative affect. J. Pain 12, 1190–1196. 10.1016/j.jpain.2011.06.008 21865092

[B107] ^*^ Salminen M. Ravaja N. (2017). The positive effects of trait emotional intelligence during a performance review discussion: a psychophysiological study. Front. Psychol. 8:463. 10.3389/fpsyg.2017.00463 28400747 PMC5368225

[B108] Salovey P. Mayer J. D. Goldman S. L. Turvey C. Palfai T. P. (1995). Emotional attention, clarity, and repair: Exploring emotional intelligence using the Trait Meta-Mood Scale, in Emotion, Disclosure and Health, ed J. W. Pennebaker (Washington, DC: American Psychological Association), 125–151. 10.1037/10182-006

[B109] ^*^ Salovey P. Stroud L. Woolery A. Epel E. S. (2002). Perceived emotional intelligence, stress reactivity, and symptom reports: further explorations using the Trait Meta Mood Scale. Psychol. Health 17, 611–627. 10.1080/08870440290025812

[B110] Salovey S. Mayer J. (1990). Emotional intelligence. Imagin. Cogn. Pers. 9,185–211. 10.2190/DUGG-P24E-52WK-6CDG

[B111] ^*^ Schneider T. R. Lyons J. B. Khazon S. (2013). Emotional intelligence and resilience. Pers. Individ. Dif. 55, 909–914. 10.1016/j.paid.2013.07.460

[B112] Schulte M. J. Ree M. J. Caretta T. R. (2004). Emotional intelligence: not much more than *g* and personality. Pers. Individ. Dif. 37, 1059–1068. 10.1016/j.paid.2003.11.014

[B113] ^*^ Schutte N. Malouff J. Simunek M. McKenley J. Hollander S. (2002). Characteristic emotional intelligence and emotional well-being. Cogn. Emotion 16, 769–785. 10.1080/02699930143000482

[B114] Schutte N. S. Malouff J. M. Hall L. E. Haggerty D. J. Cooper J. T. Golden C. J. (1998). Development and validation of a measure of emotional intelligence. Pers. Individ. Dif. 25, 167–177. 10.1016/S0191-8869(98)00001-4

[B115] ^*^ Sevdalis N. Petrides K. V. Harvey N. (2007). Trait emotional intelligence and decision-related emotions. Pers. Individ. Dif. 42, 1347–1358. 10.1016/j.paid.2006.10.012

[B116] ^*^ Singh Y. Sharma R. (2012). Relationship between general intelligence, emotional intelligence, stress levels and stress reactivity. Ann. Neurosci. 19, 107–111. 10.5214/ans.0972.7531.190304 25205980 PMC4117081

[B117] Szczygieł D. Mikolajczak M. (2017). Why are people high in emotional intelligence happier? They make the most of their positive emotions. Pers. Individ. Dif. 117, 117–181. 10.1016/j.paid.2017.05.051

[B118] Tett R. P. Freund K. A. Christiansen N. D. Fox K. E. Coaster J. (2012). Faking on self-report emotional intelligence and personality tests: effects of faking opportunity, cognitive ability, and job type. Pers. Individ. Dif. 52, 195–201. 10.1016/j.paid.2011.10.017

[B119] ^*^ Thomas L. I. Fuchs R. Klaperski S. (2018). High trait emotional intelligence in men: beneficial for perceived stress levels but disadvantageous for the physiological response to acute stressors? J. Appl. Biobehav. Res. 23:e12116 10.1111/jabr.12116

[B120] Tollenaar M. S. Elzinga B. M. Spinhoven P. Everaerd W. (2009). Immediate and prolonged effects of cortisol, but not propranolol, on memory retrieval in healthy young men. Neurobiol. Learn. Mem. 91, 23–31. 10.1016/j.nlm.2008.08.002 18761097

[B121] Van der Does H. T. D. Brink M. S. Visscher C. Huijgen B. C. H. Frencken W. G. P. Lemmink K. A. P. M. (2015). The effect of stress and recovery on field-test performance in floorball. Int. J. Sports Med. 36, 460–465. 10.1055/s-0034-1398581 25734914

[B122] Velten E. (1968). A laboratory task for induction of mood states. Behav. Res. Ther. 6, 473–482. 10.1016/0005-7967(68)90028-4 5714990

[B123] Watson D. Clark L. A. Tellegen A. (1988). Development and validation of brief measures of positive and negative affect: the PANAS scales. J. Pers. Soc. Psychol. 54, 1063–1070. 10.1037/0022-3514.54.6.1063 3397865

[B124] ^*^ Wilbraham S. J. Qualter P. Roy M. P. (2018). Emotional intelligence and cortisol responses: can laboratory findings be replicated in classrooms and using other EI measures? Pers. Individ. Dif. 120, 58–64. 10.1016/j.paid.2017.08.021

[B125] Williams C. Daley D. Burnside E. Hammond-Rowley S. (2009). Measuring emotional intelligence in preadolescence. Pers. Individ. Dif. 47, 316–320. 10.1016/j.paid.2009.03.019

[B126] Zeidner M. Matthews G. (2018). Grace under pressure in educational contexts: emotional intelligence, stress, and coping, in Emotional Intelligence in Education, eds Keefer K. Parker J. Saklofske D. (Cham: Springer), 83–110. 10.1007/978-3-319-90633-1_4

[B127] Zeidner M. Matthews G. Roberts R. (2009). What We Know About Emotional Intelligence: How it Affects Learning, Work, Relationships, and Our Mental Health. Cambridge, MA: MIT Press.

[B128] ^*^ Zysberg L. (2012). Emotional intelligence and electro-dermal activity. Appl. Psychophysiol. Biofeedback 37, 181–185. 10.1007/s10484-012-9192-3 22446999

[B129] Zysberg L. Levy A. Zisberg A. (2010). Emotional intelligence in applicant selection for care-related academic programs. J. Psychoeduc. Assess. 29, 27–38. 10.1177/0734282910365059

